# Rapid Modulation of Axon Initial Segment Length Influences Repetitive Spike Firing

**DOI:** 10.1016/j.celrep.2015.09.066

**Published:** 2015-10-29

**Authors:** Mark D. Evans, Adna S. Dumitrescu, Dennis L.H. Kruijssen, Samuel E. Taylor, Matthew S. Grubb

**Affiliations:** 1 MRC Centre for Developmental Neurobiology, King’s College London, 4^th^ Floor, New Hunt’s House, Guy’s Campus, London SE1 1UL, UK

## Abstract

Neurons implement a variety of plasticity mechanisms to alter their function over timescales ranging from seconds to days. One powerful means of controlling excitability is to directly modulate the site of spike initiation, the axon initial segment (AIS). However, all plastic structural AIS changes reported thus far have been slow, involving days of neuronal activity perturbation. Here, we show that AIS plasticity can be induced much more rapidly. Just 3 hr of elevated activity significantly shortened the AIS of dentate granule cells in a calcineurin-dependent manner. The functional effects of rapid AIS shortening were offset by dephosphorylation of voltage-gated sodium channels, another calcineurin-dependent mechanism. However, pharmacological separation of these phenomena revealed a significant relationship between AIS length and repetitive firing. The AIS can therefore undergo a rapid form of structural change over timescales that enable interactions with other forms of activity-dependent plasticity in the dynamic control of neuronal excitability.

## Highlights

• Structural plasticity at the axon initial segment can occur within hours
• Ankyrin-G and sodium channel distributions shorten after 3 hr of elevated activity
• Rapid plasticity depends on calcineurin signaling opposed by CDK5
• All else being equal, AIS shortening correlates with lowered neuronal excitability

## Graphical Abstract

**Figure:**
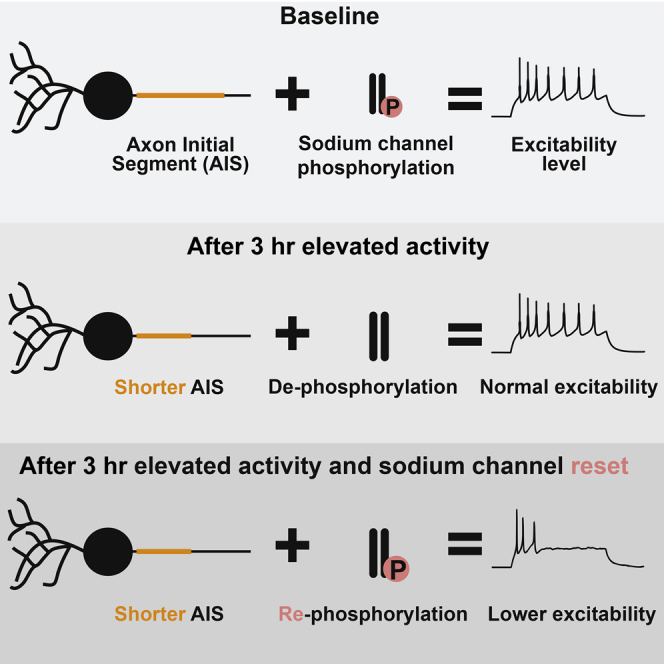

## Teaser

Evans et al. show that activity-dependent structural plasticity at the axon initial segment (AIS) can be surprisingly rapid. Elevated activity shortens the AIS in dentate granule cells after just 3 hr, an effect associated with lower excitability and reduced repetitive spiking.

## Introduction

The axon initial segment (AIS) is a molecularly defined specialization of the proximal axon that, because of its morphology, cable properties, and high density of voltage-gated sodium channels, is the site for action potential (AP) initiation (Bender and Trussell, 2012, Clark et al., 2009, Kole and Stuart, 2012). With such a crucial role in the determination of neuronal excitability, it is perhaps no surprise that the AIS is a target for considerable modulation and plasticity (Grubb et al., 2011). At sub-second timescales, AP generation and waveform properties can be influenced by AIS-localized voltage-gated sodium (Baranauskas et al., 2013, Hu et al., 2009, Kuba et al., 2006, Scott et al., 2014), potassium (Goldberg et al., 2008, Johnston et al., 2008, Kole et al., 2007, Shah et al., 2008, Shu et al., 2007), and calcium (Bender and Trussell, 2009, Bender et al., 2012, Yu et al., 2010) channels and by GABAergic synapses made by highly specialized chandelier neurons (Woodruff et al., 2010). These conductances can also be altered to control excitability over minutes-long time frames by slower, neuromodulatory mechanisms including dopamine (Bender et al., 2010, Bender et al., 2012), acetylcholine (Martinello et al., 2015), or serotonin (Cotel et al., 2013) signaling.

In addition to this short-term functional modulation of AP generation, much slower long-term mechanisms of structural plasticity can also affect excitability at the AIS. In vitro, days of chronically elevated activity can produce a distal axonal relocation of the entire AIS complex in excitatory hippocampal neurons (Evans et al., 2013, Grubb and Burrone, 2010, Muir and Kittler, 2014, Wefelmeyer et al., 2015) or trigger the opposite effect, proximal AIS relocation, in inhibitory olfactory bulb dopaminergic cells (Chand et al., 2015). In vivo, removing presynaptic input to auditory nucleus magnocellularis neurons can result in a significantly lengthened AIS after 3 days (Kuba et al., 2010). This structural plasticity at the AIS is associated with alterations in excitability (Grubb and Burrone, 2010, Kuba et al., 2010, Wefelmeyer et al., 2015) and may therefore contribute to long-term control of AP firing. However, these processes are extremely slow—their timescale of days is considerably longer than that of other structural changes that can occur locally (minutes; Harvey and Svoboda, 2007, Oh et al., 2013, Xu et al., 2009) or on a neuron-wide level (hours; Keck et al., 2011, Nägerl et al., 2004, Rocha and Sur, 1995, Yamahachi et al., 2009, Yu et al., 2011) after perturbed levels of activity. Is it possible for AIS structure to also change this quickly?

Here, we describe a rapid form of structural plasticity at the AIS that contributes, alongside other concurrent forms of intrinsic plasticity, to the regulation of neuronal excitability. Just 3 hr of patterned optogenetic stimulation or depolarization shortened the AIS of hippocampal dentate granule cells (DGCs) by 25%, with depolarization also producing a potentiation of voltage-gated sodium channel currents over the same timescale. The interplay between these two forms of calcineurin-dependent plasticity produced unchanged neuronal excitability initially after activity perturbation, but when pharmacologically isolated, rapid AIS shortening correlated significantly on a cell-by-cell basis with reduced firing of multiple APs. Structural changes at the AIS can therefore influence neuronal excitability on a rapid timescale that is directly comparable to, and permits interaction with, other well-described forms of plasticity.

## Results

### Rapid AIS Plasticity in DGCs

**Figure 1:**
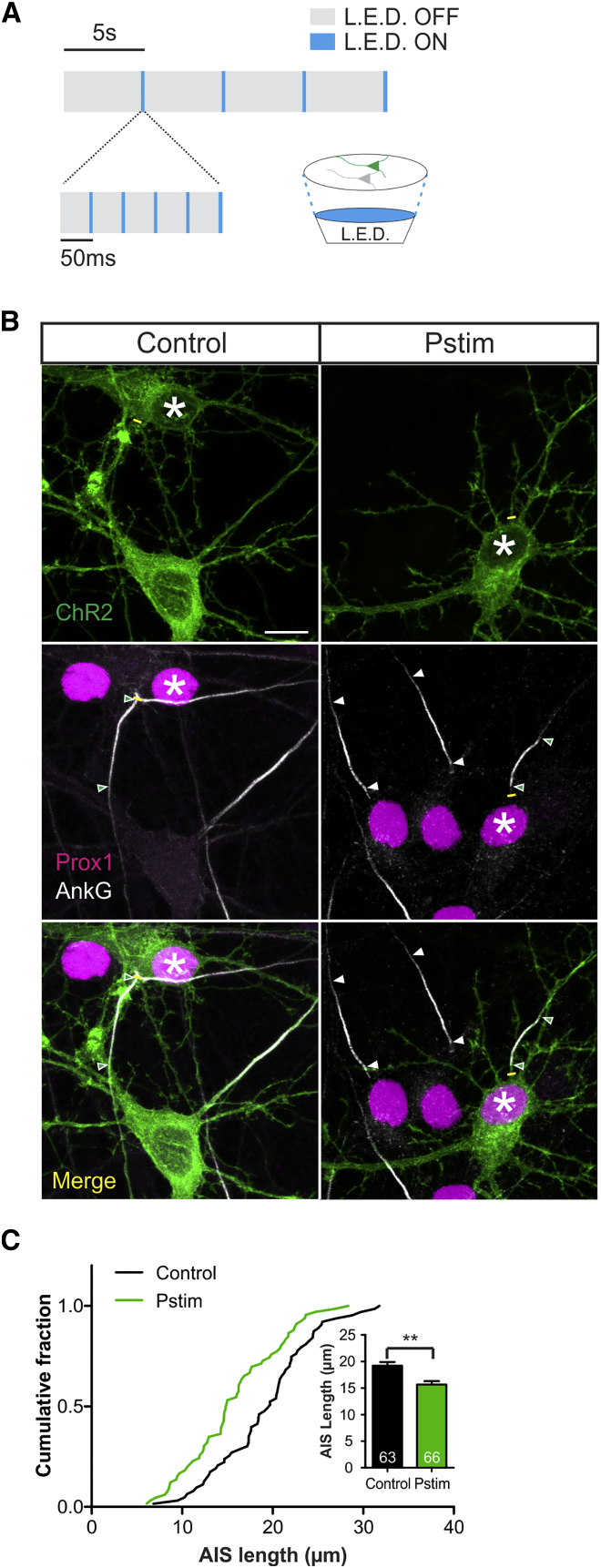
Rapid AIS Plasticity after Patterned Optogenetic Stimulation (A) Schematic of photostimulus. (B) Maximum intensity projections of sparsely ChR2-expressing cultures stained for Prox1 and AnkG after control or photostimulation (Pstim) treatment. Asterisks, soma of ChR2^+^ DGCs; lines, axon start; arrowheads, DGC AIS start and end positions; scale bar, 10 μm. (C) Cumulative fraction and (inset) mean + SEM of AIS length in ChR2^+^ DGCs. Numbers in bars, *n*; Bonferroni post-test after two-way ANOVA; ^∗∗^p < 0.01. See also Figure S1.

We initially characterized rapid changes in AIS length using optogenetics to evoke temporally structured, physiologically relevant patterns of elevated neuronal activity. Dissociated hippocampal cultures sparsely transfected with channelrhodopsin-2 (ChR2) were exposed to a structured pattern of photostimulation at 10 days in vitro (DIV; Figure 1A; Evans et al., 2013, Grubb and Burrone, 2010). Immunofluorescent labeling for the scaffolding molecule ankyrin-G (AnkG) was used to quantify AIS length in DGCs identified by prox1 expression (Evans et al., 2013, Williams et al., 2011). Just 3 hr of patterned optogenetic stimulation was sufficient to produce a significant decrease in AIS length. Photostimulated ChR2-expressing DGCs, but not their untransfected neighbors, had significantly shorter AISs than unstimulated controls (Figures 1B and 1C; transfected, control mean ± SEM 19.2 ± 0.7 μm, stimulated 15.7 ± 0.6 μm; Bonferroni post-test after two-way ANOVA, t_127_ = 3.78, p < 0.01; untransfected, control 20.1 ± 0.7 μm, stimulated 19.0 ± 0.7 μm; t_71_ = 1.22, p > 0.05). We also observed rapid shortening of the AIS in ChR2^+^ DGCs exposed to more naturalistic photostimuli, drawing inter-flash intervals from a distribution where two orthogonal parameters—frequency and ‘burstiness”—uniquely define the pattern of stimulus timing (Shimokawa et al., 2010). By systematically varying the burstiness of this distribution while keeping its overall frequency at the 1 Hz level common for DGCs in vivo (Mistry et al., 2011), we found that rapid shortening of the AIS was produced only by specific, moderately bursty temporal patterns of stimulation (Figure S1).

**Figure 2:**
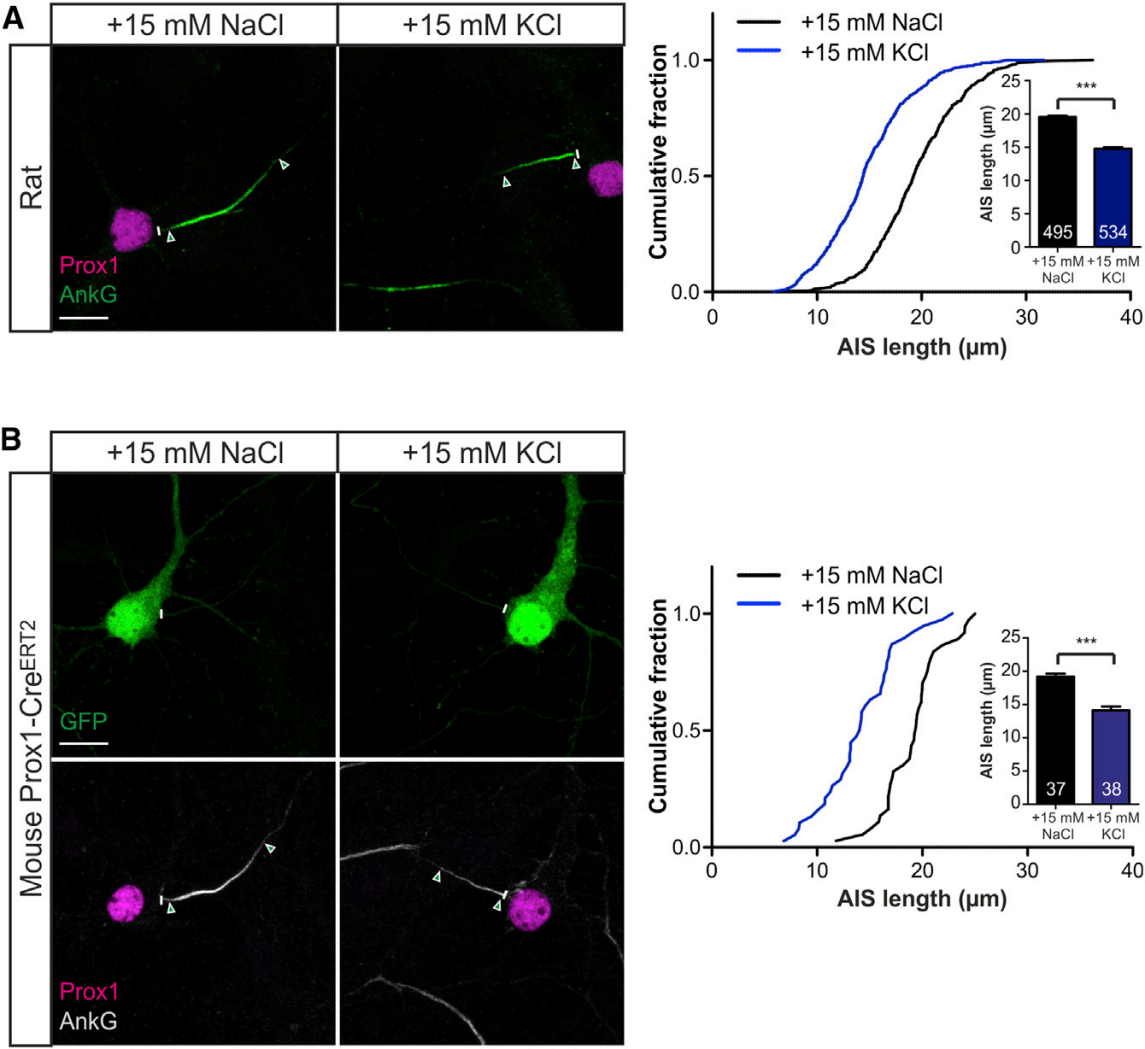
Chronic Depolarization Also Induces Rapid AIS Plasticity (A) Maximum intensity projections (left) of DGCs labeled for Prox1 and AnkG after 3-hr treatment with +15 mM NaCl or KCl. Lines, axon start; arrowheads, AIS start and end positions; scale bar, 10 μm. Plot (right) shows cumulative fraction and (inset) mean + SEM of AIS lengths. Numbers in bars, *n*; Mann-Whitney test; ^∗∗∗^p < 0.001. (B) Maximum intensity projections (left) of Prox1-CreER^T2^ DGCs infected with AAV-Flex-eGFP, labeled for Prox1 and AnkG after 3-hr treatment with +15 mM NaCl or KCl. All conventions as in (A). See also Figures S2 and S3.

Plastic alterations to AIS structure can therefore occur rapidly when induced by physiologically relevant patterns of elevated activity. To further characterize the dynamics, mechanisms, and functional consequences of this rapid AIS plasticity, we sought a less invasive stimulus that could mimic the effects of patterned photostimulation on AIS length in untransfected neurons. Chronic depolarization with elevated extracellular K^+^ is an effective stimulus for inducing voltage-dependent phenomena (Greer and Greenberg, 2008), including structural AIS plasticity (Chand et al., 2015, Evans et al., 2013, Grubb and Burrone, 2010, Muir and Kittler, 2014). By employing a range of increased [K^+^]_e_ concentrations, we found a stimulus that could reliably mimic the rapid decrease in AIS length we observed after patterned photostimulation (Figure S2A). Elevating [K^+^]_e_ by 15 mM chronically depolarized 10 DIV DGCs by ∼23 mV (+15 mM NaCl, −40.7 ± 0.8 mV; +15 mM KCl, −17.3 ± 1.5 mV), and resulted in a ∼5 μm, or ∼25%, reduction in DGC AIS length after just 3 hr (Figure 2A; +15 mM NaCl, 19.6 ± 0.2 μm; +15 mM KCl, 14.8 ± 0.2 μm; Mann-Whitney test; p < 0.0001, n = 1029).

While suggestive of rapid length changes occurring at individual DGC AISs, theoretically, this population-level effect could also have been produced by the de novo appearance of a set of prox1^+^ DGCs with short AISs and/or the disappearance of prox1^+^ cells with long AISs. However, DGC neurogenesis does not occur in our cultures (Evans et al., 2013), and cell counts ruled out any change in prox1^+^ cell density after 3-hr depolarization (+15 mM NaCl, 31.7 ± 3.0 cells/mm^2^; +15 mM KCl, 32.2 ± 4.0 cells/mm^2^; Mann-Whitney test; p = 0.64, n = 80). This does not preclude perfectly balanced, rapid changes in prox1 expression in populations of cells with very different AIS lengths. However, when we used an alternative, genetic method of DGC identification that does not rely on the stable presence of prox1 protein—cultures from *Prox1-CreER*^*T2*^ mice (Bazigou et al., 2011) treated with tamoxifen for 24 hr and infected with AAV-Flex-eGFP—we again observed ∼5 μm AIS shortening after 3-hr depolarization (10 DIV; Figure 2B; +15 mM NaCl, 19.2 ± 0.5 μm; +15 mM KCl, 14.1 ± 0.6 μm; t_73_ = 6.54; p < 0.0001). These considerations make it most likely that rapid AIS shortening is produced by changes in AIS length in individual DGCs, although time-lapse imaging experiments are required to definitively resolve this in future.

Rapid AIS shortening in rat DGCs was driven by a large proximal shift in AIS end position, with a small but significant proximal shift in AIS start position (Figure S2B). It was accompanied by a decrease in normalized total AnkG immunofluorescence (Figure S2C), suggesting a loss of AIS protein rather than compression of existing constituents into a reduced volume. Importantly, though, we also observed significant and comparable AIS shortening when we used an intensity-independent approach to quantify axonal AnkG distributions (Figure S2D–S2F). Whether induced by patterned optogenetic photostimulation or chronic depolarization, structural plasticity at the AIS can therefore occur over a rapid, hours-scale time frame.

### Cell-Type Specificity of Rapid AIS Shortening

Rapid AIS shortening did not occur in all hippocampal cell types. Using combinatorial labeling for major excitatory subtypes (Evans et al., 2013, Williams et al., 2011), we found trends toward AIS shortening in both CA1 and CA3 cells after 3-hr +15 mM KCl depolarization at 10 DIV, but a significant effect was found in CA3 neurons only (Figure S3; CA1: unpaired t test, t_86_ = 1.65, p = 0.10; CA3: unpaired t test, t_84_ = 2.44, p = 0.017). As with slower long-term AIS relocation (Evans et al., 2013), rapid AIS shortening was not observed in GABA^+^ interneurons (Figure S3; Mann-Whitney test, p = 0.37, n = 62).

### Molecular Specificity of Rapid AIS Shortening

**Figure 3:**
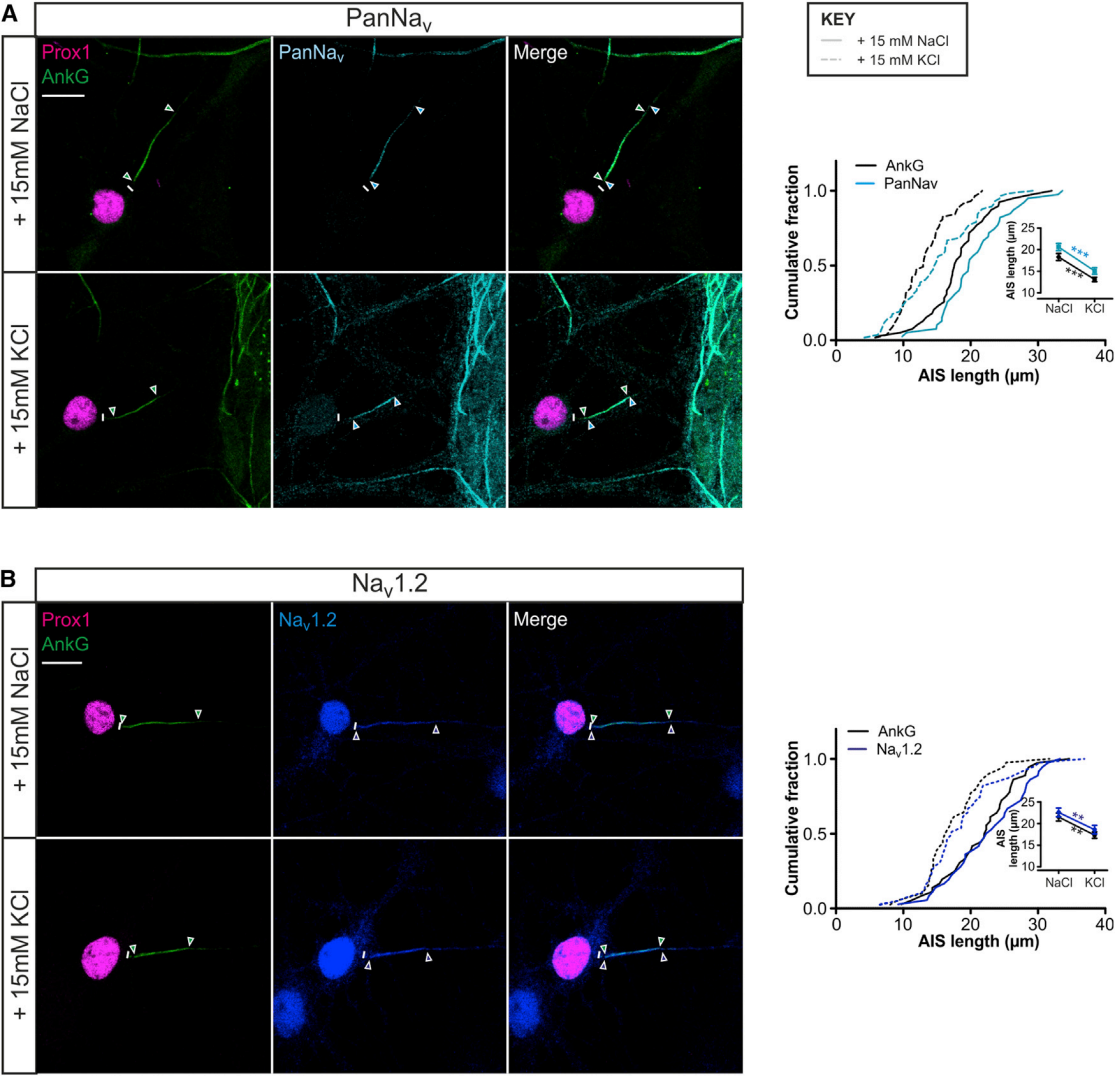
Rapid Plasticity of AIS Sodium Channel Distributions (A and B) Maximum intensity projections (left) of neurons treated for 3 hr with +15 mM NaCl or KCl, stained for AnkG and prox1, plus either pan-Na_V_ (A) or Na_V_1.2 (B). Lines, axon start; arrowheads, AIS start and end positions for AnkG (green) and sodium channels (blue); scale bar, 10 μm. Plots (right) show cumulative fraction and (inset) mean ± SEM of AIS lengths. Bonferroni post-test after two-way ANOVA; ^∗∗^p < 0.01; ^∗∗∗^p < 0.001. See also Figure S4.

To characterize the molecular changes associated with this rapid form of structural plasticity, we focused on the clear depolarization-induced decrease in rat DGC AIS length at 10 DIV. We found that the AIS distribution of all voltage-gated sodium (Na_V_) channels also shortened significantly after 3-hr depolarization (Pan-Na_V_; Figure 3A; two-way repeated-measures ANOVA; treatment, F_1,94_ = 34.6 p < 0.001; interaction, F_1,94_ = 0.11, p = 0.74). Specifically, of the major forebrain Na_V_ subunits, only Na_V_1.2 was present at the DGC AIS at this stage, where it occupied the full AIS and also shortened significantly after 3-hr depolarization (Figure 3B; two-way repeated-measures ANOVA; treatment, F_1,73_ = 10.7 p = 0.0016; interaction, F_1,73_ = 0.11, p = 0.74). The AIS distribution of neurofascin also underwent significant activity-dependent shortening, revealed by separate antibodies directed against either intracellular or extracellular epitopes (Figure S4A).

The distribution of one AIS component did not follow rapid activity-dependent AnkG changes, however. The unidentified microtubule-associated protein labeled by the “pIκBα” antibody (Buffington et al., 2012) is present along the full length of the DGC AIS and correlates well with AnkG under control conditions, but unlike AnkG in the same neurons, its distribution was unchanged after 3-hr depolarization (Figure S4B). This suggests that rapid AIS shortening may involve a detachment of AnkG-binding components from the underlying cytoskeleton.

### Rapid AIS Shortening Is Not an Injury Response

**Figure 4:**
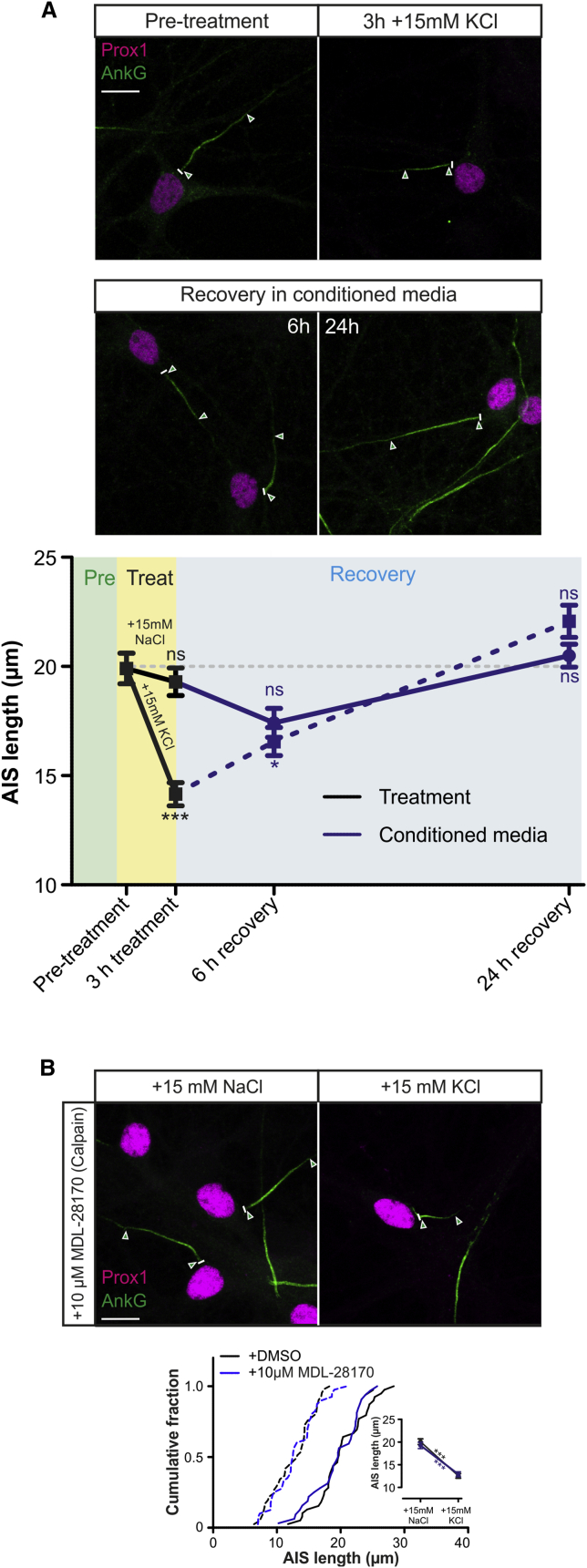
Rapid AIS Plasticity Is Not an Injury Response (A) Maximum intensity projections (top) of neurons labeled for AnkG and prox1 before and after treatment with 3 hr +15 mM KCl. Lines, axon start; arrowheads, AIS start and end positions; scale bar, 10 μm. Plot (bottom) shows mean ± SEM of AIS length. Dunn’s post-test versus pre-treatment after Kruskal-Wallis ANOVA ^∗^p < 0.05; ^∗∗∗^p < 0.001; ns, non-significant. (B) Maximum intensity projections (top) of neurons labeled for AnkG and prox1 following 3 hr +15 mM NaCl or KCl treatment with the calpain inhibitor MDL-28170. All conventions as in (A). Bonferroni post-test after two-way ANOVA; ^∗∗∗^p < 0.001.

A well-characterized AIS response to injury involves calpain-dependent irreversible loss of the entire structure (Schafer et al., 2009). In contrast, rapid AIS shortening proved to be fully reversible and independent of calpain signaling. After 3-hr depolarization, we returned our rat neurons to control media, observing a trend toward AnkG-defined AIS length recovery after 6 hr and complete recovery after 24 hr (Figure 4A; Dunn’s post-test versus pre-treatment following Kruskal-Wallis one-way ANOVA; 6 hr recovery, p < 0.05; 24 hr recovery, p > 0.05). Also, the calpain inhibitor MDL-28170 had no effect on DGC AIS length in either control or depolarized conditions (Figure 4B; two-way ANOVA; interaction, F_1,148_ = 0.94, p = 0.33).

### Rapid AIS Shortening Is Mediated by Calcineurin and Opposed by CDK5 Signaling

**Figure 5:**
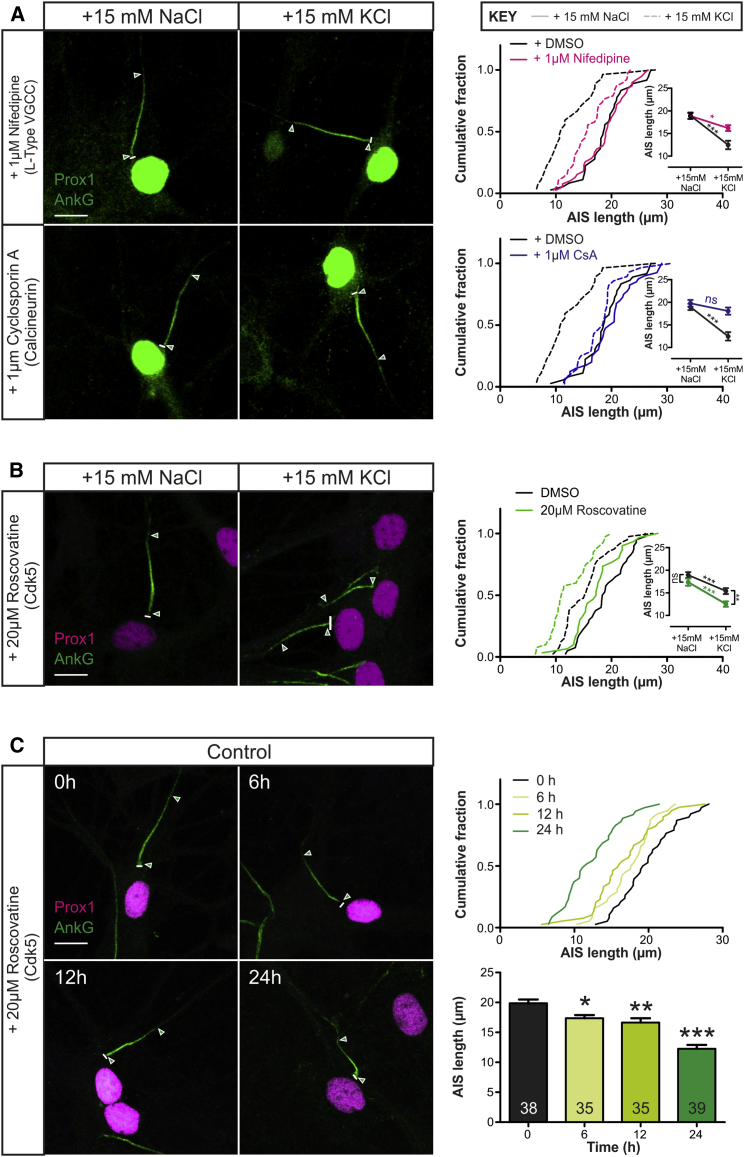
Rapid AIS Plasticity Depends on Calcineurin and Is Opposed by CDK5 Signaling (A) Maximum intensity projections (left) of neurons labeled for AnkG and prox1 with a common secondary antibody following 3 hr +15 mM NaCl or KCl treatment with the Ca_V_1 blocker nifedipine or the calcineurin inhibitor cyclosporin A (CsA). Lines, axon start; arrowheads, AIS start and end positions; scale bar, 10 μm. Plots (right) show cumulative fraction and (inset) mean ± SEM of AIS length. Bonferroni post-test after two-way ANOVA; ^∗^p < 0.05; ^∗∗∗^p < 0.001; ns, non-significant. (B) Maximum intensity projections (left) of neurons labeled for AnkG and prox1 after 3 hr +15 mM NaCl or KCl treatment with the CDK5 blocker roscovitine. Bonferroni post-test after two-way ANOVA; ^∗∗^p < 0.01; all other conventions as in (A). (C) Maximum intensity projections (left) of neurons labeled for AnkG and prox1 after different durations of roscovitine treatment. Plots (right) show cumulative fraction (top) and mean + SEM (bottom) of AIS length. Numbers in bars, *n*; Tukey post-test versus 0 hr after 1-way ANOVA; ^∗^p < 0.05; ^∗∗^p < 0.01; ^∗∗∗^p < 0.001. See also Figure S5.

APs are not required for rapid AIS plasticity in rat DGCs: blocking Na_V_ with 1 μM tetrodotoxin (TTX) had no effect on the shortened AnkG distribution produced by 3-hr depolarization (Figure S5A). Instead, rapid AIS shortening depends on signaling through L-type Ca_V_1 calcium channels, with 1 μM nifedipine significantly reducing the effect of 3-hr depolarization on AIS length (Figure 5A; two-way ANOVA; interaction, F_1,136_ = 6.80, p = 0.010). This reduction was marked but not absolute; a small but significant shortening response remained in the presence of Ca_V_1 inhibition (Figure 5A; Bonferroni post-test, p < 0.05). This contrasts with the complete dependence of AIS relocation on L-type signaling (Evans et al., 2013) and suggests that the slightly stronger +15-mM KCl stimulus required for reliable shortening after 3 hr (Figure S2) acts through additional voltage-dependent pathways. These additional pathways do not involve the recruitment of depolarization-evoked synaptic signaling, however, because blocking glutamatergic receptors with and without spike blockade, or blocking GABAergic signaling, had no effect on the shortening produced by 3-hr depolarization (Figure S5A).

Slow activity-dependent AIS relocation depends on the Ca^2+^-activated phosphatase calcineurin (Evans et al., 2013), and this is also true for rapid AIS shortening. Blocking calcineurin with 1 μM cyclosporin (CsA) prevented AIS shortening after 3-hr depolarization (Figure 5A; two-way ANOVA; interaction, F_1,126_ = 9.05, p = 0.003), leaving a small (<2 μm) decrease in AIS length that was no longer statistically significant (Bonferroni post-test, p > 0.05) or carried by CaMKII signaling (Figure S5A).

We next investigated the potential role in rapid AIS shortening of signaling molecules known to influence the development of the structure. Blocking casein kinase 2 (Bréchet et al., 2008) with 1 μM TBB (4,5,6,7-tetrabromo-1*H*-benzotriazole) had no effect on depolarization-induced AIS shortening (Figure S5A). However, blocking cyclin-dependent kinase 5 (CDK5; Trunova et al., 2011) with 20 μM roscovitine produced a trend toward AIS shortening after 3 hr in baseline conditions and significantly augmented the shortening response to 3-hr depolarization (Figure 5B; two-way ANOVA; treatment, F_1,145_ = 39.3, p < 0.0001; drug, F_1,145_ = 11.10, p = 0.0011; interaction, F_1,145_ = 0.94, p = 0.33; Bonferroni post-test for DMSO versus roscovitine in +15 mM NaCl, p > 0.05; in +15 mM KCl, p < 0.01), suggesting that this kinase acts both constitutively and in an activity-dependent manner to oppose reductions in AIS length. Indeed, CDK5 block alone was sufficient to significantly reduce the length of the AIS after 6 hr, with greater effect after 12 and 24 hr (Figure 5C; Tukey’s post-test versus 0 hr after one-way ANOVA; 6 hr, p < 0.05; 12 hr, p < 0.01; 24 hr, p < 0.0001). Importantly, this effect of CDK5 blockade was not altered by 1 μM nifedipine (Figure S5B), demonstrating that it did not act indirectly via neuronal depolarization and/or Ca_V_1 activation. It was, however, partially reversed by concurrent treatment with 1 μM CsA (Figure S5B). Together with the additive effects of 3-hr depolarization and roscovitine (Figure 5B), this suggests that CDK5 and calcineurin partially interact in a common pathway: elevated neuronal activity reduces AIS length via a calcineurin-driven process that does not depend on CDK5 inhibition, but constitutive CDK5 signaling maintains AIS length in part by counteracting calcineurin activity.

### Rapid AIS Shortening Dampens Neuronal Excitability But Can Be Offset by Na_V_ Modulation

**Figure 6:**
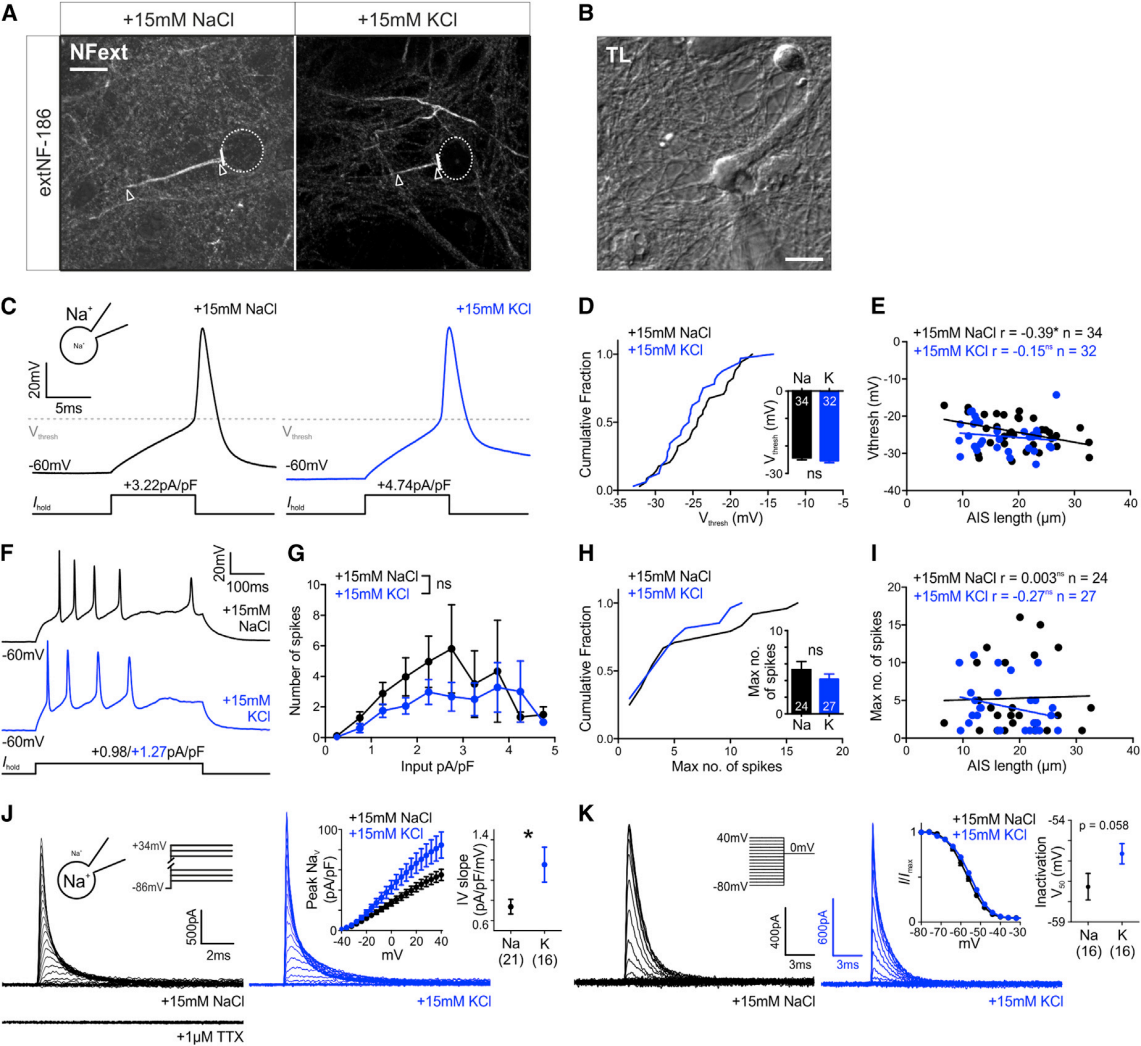
Functional Effects of 3-hr Depolarization (A) Maximum intensity projections of neurons treated for 3 hr with +15 mM NaCl or KCl, live-labeled for neurofascin (NF-ext). Dotted lines, soma; lines, axon start; arrowheads, AIS start and end position; scale bar, 10 μm. (B) Transmitted light image of the +15 mM NaCl cell from (A) targeted for whole-cell recording. Scale bar, 10 μm. (C) Example whole-cell current-clamp recordings of threshold APs fired to 10-ms somatic current injection. (D) Cumulative fraction and (inset) mean − SEM plots of voltage threshold. Numbers in bars, *n*. t test; ns, non-significant. (E) Scatterplot of AIS length versus voltage threshold. Each dot, one cell; lines, best-fit linear regression; Pearson correlation; ^∗^p < 0.05; ns, non-significant. (F) Example whole-cell current-clamp recordings of maximum spike number fired to 500-ms somatic current injection. (G) Mean ± SEM spike number produced by different amplitude 500-ms somatic current injection. Mixed model effect of treatment; ns, non-significant. (H) Cumulative fraction and (inset) mean + SEM plot of maximum spike number fired to 500-ms somatic current injection. Mann-Whitney test; ns, non-significant. Numbers in bars, *n*. (I) Scatterplot of AIS length versus maximum spike number. Each dot, one cell; lines, best fit linear regression; Spearman correlation; ns, non-significant. (J) Example whole-cell sodium current activation under reversed ionic gradient, blocked with TTX (bottom). Insets show mean ± SEM sodium current amplitude at different voltages (left) and mean ± SEM slope of this relationship (right). Numbers in brackets, *n*; t test with Welch’s correction; ^∗^p < 0.05. (K) Example whole-cell sodium current steady-state inactivation. Insets show mean ± SEM normalized current at different voltages (left) and mean ± SEM. V_50_ (right). Numbers in brackets, *n*. p value, t test. See also Figure S6 and Table S1.

All else being equal, a reduced distribution of Na_V_ channels in the proximal axon (Figure 3) should lower neuronal excitability (Baranauskas et al., 2013, Kaphzan et al., 2011, Kaphzan et al., 2013, Kress et al., 2010, Kuba et al., 2010, Kuba et al., 2014, Wang et al., 2011). We tested this prediction by obtaining whole-cell patch-clamp recordings from 10- to 12-DIV rat DGCs in which the length of the AIS could be determined using rat-specific live immunolabel for neurofascin (Figures 6A and 6B; Schafer et al., 2009). After 3-hr treatment with +15 mM NaCl or KCl, cells were recorded in identical extracellular solution to assess their AP properties following somatic current injection. Surprisingly, the features of single spikes fired to a threshold 10-ms current pulse were no different between control and depolarized groups (Figures 6C and 6D; Table S1), despite a significant depolarization-induced drop in input resistance (Evans et al., 2013, Grubb and Burrone, 2010, O’Leary et al., 2010; Table S1; Mann-Whitney test, p = 0.044, n = 68) that produced a strong trend toward elevated rheobase in the +15-mM KCl group (Table S1; Mann-Whitney test; p = 0.073, n = 32). Neither were any spike waveform properties correlated with live-labeled AIS length, aside from a weak relationship with voltage threshold in the control group only (Figure 6E; Table S1; Pearson r = −0.39, p = 0.022, n = 34). A trend toward lower spike output to prolonged 500-ms current injections in the depolarized, AIS-shortened group was not significant either (Figures 6F–6I; Table S1).

This lack of predicted effect of rapid AIS shortening led us to ask whether 3-hr depolarization was in fact associated with a functional decrease in available Na_V_ channels. To minimize clamp errors due to runaway depolarization, we used a reversed [Na^+^] gradient that produced well-controlled outward sodium currents in whole-cell voltage-clamp mode (Figure 6J; Garrido et al., 2003) and found that 3-hr depolarization was in fact associated with a significant *increase* in whole-cell Na_V_ current (Figure 6J; t test with Welch’s correction; t_20_ = 2.2, p = 0.038). In the on-cell configuration, however, we saw no increase in somatic Na_V_ current (Figures S6A and S6B).

What could be producing this effect? Na_V_ channels, particularly the Na_V_1.2 subtype (Figure 3), are modulated by calcineurin and protein phosphatase 1 (PP1) dephosphorylation that directly increases channel current (Cantrell and Catterall, 2001, Li et al., 1992). Given the requirement for calcineurin signaling in rapid AIS shortening (Figure 5), could our depolarizing stimulus also be modulating Na_V_ current? Channel kinetics gave some indication that this might be the case: Na_V_ dephosphorylation by calcineurin and its opposing re-phosphorylation by protein kinase A (PKA) produce a ∼3-mV shift in inactivation V_50_ (Cantrell et al., 1999, Li et al., 1992). This minor kinetic change accounts for <10% of current amplitude modulation (Cantrell et al., 1999) but is a hallmark signature of this form of Na_V_ modification and was indeed present in our recordings (Figure 6K; t test, t_30_ = 1.97, p = 0.058). In contrast, Na_V_ activation curves were unaffected by 3-hr depolarization (V_50_: +15 mM NaCl −20.1 ± 1.8 mV; +15 mM KCl −20.1 ± 1.3 mV; Mann-Whitney test; p = 0.71, n = 37; Slope factor: +15 mM NaCl 12.0 ± 0.5; +15 mM KCl 11.2 ± 0.5; t_35_ = 1.15, p = 0.26).

**Figure 7:**
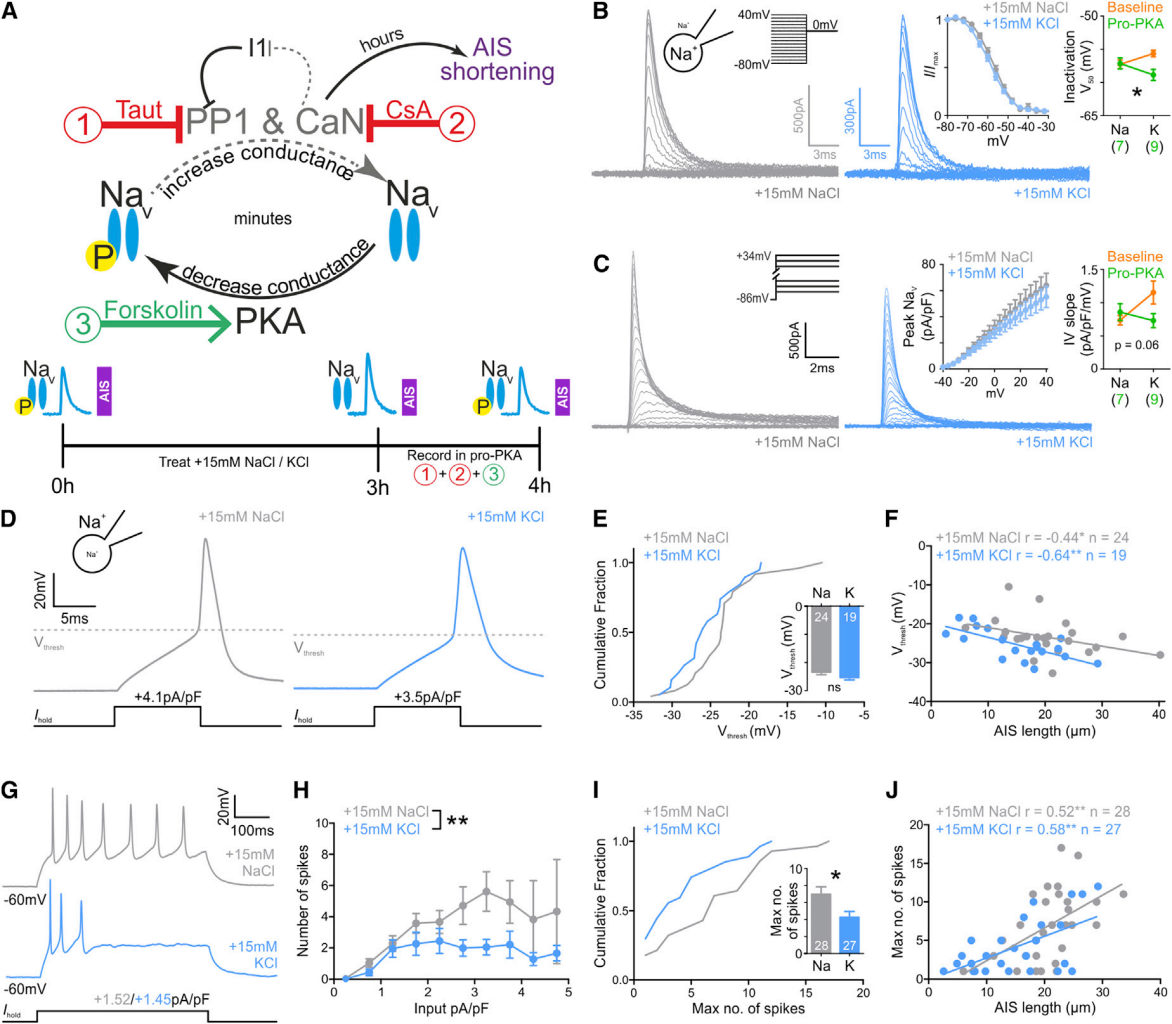
Pharmacologically Isolated Rapid AIS Plasticity Influences Multiple Spike Firing (A) Diagram of pro-PKA treatment rationale and timeline. CaN, calcineurin; Taut, tautomycetin; CsA, cyclosporin A. (B) Example whole-cell sodium current steady-state inactivation under pro-PKA conditions after 3 hr +15 mM NaCl or KCl treatment. Insets show mean ± SEM normalized current at different voltages in both pro-PKA groups (left) and mean ± SEM. V_50_ for all groups (right). Numbers in brackets, *n* in each pro-PKA group; interaction in two-way ANOVA; ^∗^p < 0.05. (C) Example whole-cell sodium current activation in pro-PKA conditions. Insets show mean ± SEM sodium current amplitude at different voltages in each pro-PKA treatment group (left) and mean ± SEM slope for all groups (right). Numbers in brackets, *n* in each pro-PKA group. p value, interaction in two-way ANOVA. (D) Example whole-cell current-clamp recordings of threshold APs fired in pro-PKA conditions to 10-ms somatic current injection. (E) Cumulative fraction and (inset) mean − SEM plots of voltage threshold in both pro-PKA groups. Numbers in bars, *n*. Mann-Whitney test; ns, non-significant. (F) Scatterplot of AIS length versus voltage threshold for both pro-PKA groups. Each dot, one cell; lines, best-fit linear regression; Spearman correlation, ^∗^p < 0.05; ^∗∗^p < 0.01. (G) Example whole-cell current-clamp recordings of maximum spike number fired in pro-PKA conditions to 500-ms somatic current injection. (H) Mean ± SEM spike number produced by different amplitude 500-ms somatic current injection in each pro-PKA group. Mixed model effect of treatment; ^∗∗^p < 0.01. (I) Cumulative fraction and (inset) mean + SEM plot of maximum spike number fired to 500-ms somatic current injection in each pro-PKA group. Numbers in bars, *n*; t test; ^∗^p < 0.05. (J) Scatterplot of AIS length versus maximum spike number for both pro-PKA groups. Each dot, one cell; lines, best-fit linear regression; Pearson correlation; ^∗∗^p < 0.01. See also Figure S7 and Table S2.

If the functional effects of rapid AIS shortening were being offset by calcineurin-dependent Na_V_ modulation, we reasoned that we might be able to reveal the former by pharmacologically reversing the latter. Exploiting the rapid modulation of Na_V_ channels and the much slower recovery of AIS plasticity (Figure 4), we used a “pro-PKA” cocktail to minimize calcineurin and PP1 signaling while boosting PKA activity in the hour immediately following 3-hr depolarization (Figure 7A). This should rapidly re-phosphorylate Na_V_ and thereby re-set the potentiation of whole-cell Na_V_ current, without affecting depolarization-induced AIS shortening (Figure 7A). Indeed, pro-PKA treatment did not significantly change AIS length (Figures S7A and S7B), but it did reverse the depolarization-induced changes in Na_V_ channel properties. Inactivation V_50_ was now significantly repolarized (Figure 7B; two-way ANOVA; interaction, F_1,44_ = 5.44, p = 0.024), and whole-cell Na_V_ current amplitudes now trended toward decreased levels after depolarization (Figure 7C; two-way ANOVA; interaction, F_1,49_ = 3.70, p = 0.060).

With Na_V_ modulation pharmacologically reset, we now saw functional correlates of rapid AIS shortening. No single spike parameters differed between control and depolarized neurons in pro-PKA conditions (Table S2), but there were significant negative correlations between voltage threshold and AIS length in both treatment groups (Figures 7D–7F; Table S2; +15 mM NaCl, Spearman r = −0.44, p = 0.03, n = 24; +15 mM KCl, Spearman r = −0.64, p = 0.003, n = 19). AIS shortening was therefore associated with an increase in the depolarization required to fire a single spike, although an opposing AIS-independent effect of depolarization obviated any group differences on this measure (Figures 7D–7F; Table S2; Mann-Whitney test, p = 0.14, n = 43).

The effects of rapid AIS shortening were much clearer on repetitive spike firing in pro PKA conditions, where 3 hr depolarization produced a significant decrease in the DGC input-output function (Figures 7G and 7H; treatment in repeated-measures mixed model, F_1,43_ = 7.49, p = 0.008) with a significant reduction in maximum firing frequency (Figure 7I; t test, t_53_ = 2.43, p = 0.019). These effects were not caused by alterations in intrinsic membrane properties or tonic DGC responses to depolarizing current (Figures S7C–S7M), nor could they be explained by changes in somatic potassium channels (Figures S6C–S6H; Misonou et al., 2004). Instead, maximum spike number under pro-PKA conditions correlated positively and significantly with live-labeled AIS length in both control and depolarized DGCs (Figure 7J; Table S2; +15 mM NaCl Pearson r = 0.52, p = 0.005, n = 28; +15 mM KCl, Pearson r = 0.58, p = 0.001, n = 27). Overall, our data demonstrate that rapid AIS shortening is associated with dampened excitability in multiple spike firing but that its effects can be offset by concurrent modulation of Na_V_ channel properties.

## Discussion

Structural plasticity at the AIS can be surprisingly rapid, with just 3 hr of patterned optogenetic photostimulation or depolarization sufficient to significantly reduce AIS length. This activity-dependent shortening is cell-type specific and fully reversible, involves a reduction in the axonal distribution of Na_V_ channels, and depends on calcineurin signaling. Functionally, rapid AIS shortening is associated with a significant dampening of neuronal excitability, an effect offset under particular stimulus conditions by coincident calcineurin-dependent modulation of Na_V_ channel properties.

Activity-dependent shortening is the most rapid form of structural change known to occur at the AIS. Previous studies observed AIS plasticity after days of altered neuronal activity (Chand et al., 2015, Evans et al., 2013, Grubb and Burrone, 2010, Kuba et al., 2010, Kuba et al., 2014, Muir and Kittler, 2014, Wefelmeyer et al., 2015), described structural AIS changes weeks after pathological manipulations (Baalman et al., 2013, Harty et al., 2013, Hinman et al., 2013), or found AIS abnormalities in animals with congenital diseases (Harty et al., 2013, Kaphzan et al., 2011, Kaphzan et al., 2013). A potential exception is the calpain-dependent and irreversible loss of the AIS after injury (Schafer et al., 2009), but this study employed a ∼24-hr delay between hours-scale manipulations and assessments of AIS integrity. A timescale of hours rather than days now places activity-dependent AIS change squarely within the time frame occupied by many other forms of neuronal plasticity, particularly those involving structural alterations (Keck et al., 2011, Lei et al., 2006, Nägerl et al., 2004, Rocha and Sur, 1995, Sin et al., 2002, Yamahachi et al., 2009, Yu et al., 2011). Structural changes at the AIS may therefore occur alongside, and interact with, many other well-described forms of activity-dependent neuronal plasticity.

Indeed, we show here that coincident activity-dependent phenomena can interact to control neuronal excitability. Over a period of hours, AIS shortening and Na_V_ modulation, both driven by calcineurin signaling but opposed by different kinases, have the net effect of keeping repetitive firing unchanged (Figure 6). Why might such a situation occur? Perhaps the activation requirements for these two opposing forms of plasticity are largely separate, with physiological responses rarely producing the limited set of activity patterns that can trigger both effects. Future work should address this possibility; for instance, naturalistic stimulation of DGCs (Figures 1 and S1) may induce AIS shortening without concomitant Na_V_ modulation. Alternatively, co-activation of these two opposing plasticities could be adaptive, representing a coordinated response that maintains a given operating range of cellular excitability.

Additional synergy between different forms of plasticity is suggested by the fact that hours-scale AIS shortening precedes slower, days-scale AIS relocation, and that both processes rely on a common Ca_V_1 and calcineurin signaling pathway (Evans et al., 2013, Grubb and Burrone, 2010). There may be a continuum of AIS plasticity, with initial shortening superseded by relocation if calcineurin signaling is elevated for a sufficient period. Shortening could therefore be a precursor to relocation—perhaps the AIS must decrease in density (Figure S2) and decouple from the cytoskeleton (Figure S4) before it can relocate along the axon. Alternatively, rapid shortening may have independent functional consequences that are superseded by the effects of subsequent relocation (Baranauskas et al., 2013, Grubb and Burrone, 2010, Muir and Kittler, 2014, Wefelmeyer et al., 2015).

Indeed, when dissociated from coincident Na_V_ modulation, we found that AIS shortening is associated with a decrease in neuronal excitability on measures of multiple, but not single, AP firing (Figure 7). Such preferential effects on spike frequency may have functional relevance, given that multiple, but not single, DGC APs are effective in driving postsynaptic spikes in CA3 cells (Henze et al., 2002) and that multiple DGC spiking is crucial for LTP at this connection (Kobayashi and Poo, 2004, Mistry et al., 2011). Nevertheless, data (Kaphzan et al., 2011, Kaphzan et al., 2013, Kuba and Ohmori, 2009, Kuba et al., 2010) and models (Baalman et al., 2013, Kress et al., 2010, Kuba et al., 2006, Wang et al., 2011) predict that AIS shortening should also cause changes in single spike parameters. We envisage two scenarios, however, in which multiple firing could be preferentially affected by AIS shortening:

(1) A separate, AIS-dependent mechanism for reducing repetitive spiking. AIS K_V_2.2 channels are a candidate, since they preferentially control multiple spiking via their effects on the inter-spike afterhyperpolarization (AHP) (Johnston et al., 2008). However, our data found no correlation between AIS length and this parameter (Table S2). Instead, given that APs initiate near the distal end of the DGC AIS (Schmidt-Hieber et al., 2008), a shorter AIS may move spike initiation closer to the soma and make it more susceptible to depolarization-induced Na_V_ inactivation during high-frequency firing (Kuba et al., 2006, Scott et al., 2014).
(2) AIS shortening affects both repetitive and single spiking but has a more marked effect on multiple firing. This possibility has some support from our data, where there are correlations in the predicted direction between AIS length and several single-spike parameters (e.g., V_thresh_, V_max_, Max dVdt; Table S2; Baalman et al., 2013, Kaphzan et al., 2011, Kaphzan et al., 2013, Kuba and Ohmori, 2009, Kuba et al., 2010), but the strength and slope of these relationships are insufficient to produce significant group differences.

We find that rapid AIS shortening depends on calcineurin and is opposed by CDK5 (Figure 5), in common with other forms of plasticity where these two pathways work in opposition. At the presynaptic terminal, CDK5 facilitates and calcineurin inhibits vesicle exocytosis (Kim and Ryan, 2013). In hippocampal neurons, these pathways influence neuronal excitability by modifying clustering of somatic K_V_2.1 channels (Cerda and Trimmer, 2011, Misonou et al., 2004). Although our data suggest that K_V_2.1 modulation cannot account for the reduced excitability associated with AIS shortening (Figure S6), signaling through common calcineurin and CDK5 pathways may co-regulate these distinct somatic and axonal, minutes- and hours-scale processes to sequentially control neuronal excitability. Overall, given the plethora of activity-dependent Hebbian and homeostatic mechanisms known to operate with potential spatial, temporal, and molecular overlap in individual cell types, future efforts will need to focus on their potential synergistic and antagonistic interactions in determining neuronal function.

## Experimental Procedures

### Dissociated Culture

Humane killing for tissue collection conformed to local King’s College London ethical approval under the UK Supplementary Code of Practice, The Humane Killing of Animals under Schedule 1 to the Animals (Scientific Procedures) Act 1986. We dissected hippocampi from embryonic day 18 (E18) Wistar rat embryos or E17 *Prox1-CreER*^*T2*^ × C57Bl6J mice (Bazigou et al., 2011; Charles River Laboratories) of either sex into Hank’s balanced salt solution (HBSS). Tissue was digested with trypsin (Worthington, 0.5 mg/ml; 15 min [rat] or 4 min [mouse] at 37°C) before trituration and plating at 45,000 (rat) or 100,000 (mouse) cells per well on 13-mm glass coverslips pre-coated with poly-l-lysine (50 μg/ml; Sigma) and laminin (40 μg/ml). Neurons were cultured at 37°C with 5% CO_2_ in Neurobasal medium plus 1% B27, 1% fetal calf serum, and 500 μM Glutamax. Prox1-CreER^T2^ cultures were treated with 1 μM 4-OH-tamoxifen (Sigma) for 24 hr at 1 DIV and infected with 1:10,000 AAV-Flex-eGFP (Penn Vector Core AV-9-ALL854) continually from 1 DIV. At 4 DIV, media was half-changed with Neurobasal plus 2% B27 and 500 μM Glutamax. At 7 DIV, media was made up to 1 ml by adding 400 μl Neurobasal with 2% B27 and 500 μM Glutamax. We transfected pLenti-Synapsin-hChR2(H134R)-EYFP-WPRE (optogenetics.org) at 7 DIV using lipofectamine2000. Unless otherwise stated, all culture reagents were from Invitrogen.

### Photostimulation, Depolarization, and Pharmacology

At 10 DIV, neurons were prepared for photostimulation by supplementing their media for ≥2 hr with an antioxidant cocktail (3.2 μM glutathione, Fisher Scientific; 110 μM vitamin C, 100 μM Trolox, 2.3 μM vitamin E, 77 nM superoxide dismutase, 10 nm catalase; all Sigma) and blocking AMPA-mediated synaptic transmission with NBQX (Sigma). 4-well plates containing treated media and neurons were then placed on top of Royal Blue LEDs (Luxeon Star) with individual collimators (RS Components), on an aluminum heat sink (Fisher Electronik), driven by DC/DC LED drivers (RS Components) controlled by in-house software (Andrew Lowe). For structured patterns of photostimulation, flashes of 5 ms duration were grouped into bursts of 5 flashes at 20 Hz, with one burst every 5 s (Figure 1A). For naturalistic patterned photostimulation, flashes of 5 ms duration were separated by inter-flash intervals drawn at random from a discrete negative binomial distribution with mean 1,000 ms (1 Hz overall frequency) and different CV^2^ (burstiness) values (Shimokawa et al., 2010). We generated hour-long sequences for each burstiness using custom-written Matlab routines (M.S.G. and Nick Lesica, UCL Ear Institute, UK), then looped these for a total of 3 hr of photostimulation.

For depolarization, neurons were treated in fully conditioned media at 10 DIV with stated concentrations of KCl or NaCl as osmolarity control. For recovery, after 3-hr depolarization, cells were placed back into conditioned media. Drugs were made up as per the manufacturers’ instructions and added to neurons at previously described effective working concentrations 1 hr before treatment.

### Immunocytochemistry

Neurons were fixed in 4% paraformaldehyde (PFA; TAAB Laboratories; in 3% sucrose, 60 mM PIPES, 25 mM HEPES, 5 mM EGTA, 1 mM MgCl_2_) for 20 min at room temperature (RT), then washed in PBS. We permeabilized for 5 min with 0.25% Triton X-100 (Sigma) before blocking for 1 hr in 10% goat serum (GS; Sigma). Coverslips were placed in primary antibody (Table S3) solution for 90 min in 2% GS, washed, then placed in relevant secondary antibody solution (Invitrogen Alexa Fluor-conjugated, in 2% GS, all 1:1,000) for 1 hr, washed, and mounted in MOWIOL (Calbiochem). As exceptions, anti-Na_V_1.2 required 1% PFA fixation, and anti-Pan-Na_V_ required 3 min post-fixation incubation in 0.2 mg/ml pepsin (in 0.2 M HCl, Dako).

For cell types, DGCs were identified by the presence of nuclear prox1 immunofluorescence, CA1 pyramidal cells by the presence of nuclear CTIP2, but not prox1, and CA3 pyramidal cells by neuron-wide expression of αCaMKII in the absence of both nuclear prox1 and CTIP2.

### Imaging and Analysis

All imaging and analysis was performed blind to experimental group. Neurons with AISs of somatic origin were visualized under epifluorescence and imaged using a laser scanning confocal microscope (Zeiss LSM 710) with appropriate excitation and emission filters, 1 AU pinhole, and 40× oil (fixed) or water (live) immersion objectives. Laser power and gain settings were adjusted to prevent signal saturation. Images were taken with 3× zoom, 512 × 512 pixels (0.138 μm/pixel) in z stacks with 0.5-μm steps. Stacks were imported into Matlab (Mathworks) for analysis using custom written functions (freely available at Matlab Central). We drew a line profile along each maximum intensity projection starting at the soma, down the axon, through and past the AIS. In live-labeled images, high background levels required AIS start and end positions to be determined by eye along this line profile. In fixed images, at each pixel in the axonal profile, fluorescence intensity values were averaged over a 3 × 3-pixel square centered on the pixel of interest. Averaged profiles were smoothed using a 40-point (∼5 μm) sliding mean and normalized between 1 and 0. AIS start and end positions were the proximal and distal axonal positions where the normalized, smoothed profile declined to 0.33. AIS length was calculated as the axonal distance between start and end positions.

For a subset of images with high signal:noise, we employed a semi-automated approach to measure AIS length (Figures S2C–S2E). The image was smoothed with a 2D Gaussian (20 × 20 pixel window, Gaussian SD = 2 pixels), then thresholded at 11% of the maximum intensity pixel across the entire image. The image was then morphologically opened and closed using a 3 × 3 pixel uniform structural element. After user determination of the AIS start point, connected components of the binary image were calculated and the single connected element closest to the AIS start isolated. This element was thinned to produce an “AIS skeleton” 1 pixel wide, the length of which was determined by fitting a 2D cubic smoothing spline to the ordered point cloud of AIS skeleton pixels.

### Electrophysiology

Recordings were obtained by experimenters blind to treatment group, from 10- to 12-DIV DGCs identified by their distinct morphology (Evans et al., 2013). Pipettes were pulled from borosilicate glass (outer diameter 1.5 mm, inner diameter 1.17 mm, Harvard Apparatus; 3–7 MΩ). Recordings were obtained at RT (unless stated otherwise) with a Heka EPC10/2 amplifier coupled to Patchmaster software. Signals were Bessel filtered at 10 kHz (filter 1), and 2.9 kHz (filter 2; active filters employed in voltage clamp only), digitized, and sampled at 20–200 kHz (5- to 50-μs sample interval). Fast capacitance was compensated in the on-cell configuration. After membrane rupture, at a holding voltage of −60 mV (uncorrected, as with all voltages in this study, for calculated liquid junction potentials (LJPs) associated with each combination of extra- and intracellular solutions; see below), and with slow capacitance compensation inactive, we used responses to 10-mV hyperpolarization to estimate series resistance (*R*_s_; < 25 MΩ for all cells), membrane resistance (*R*_m_), and membrane capacitance (*C*_m_). Recordings where *R*_s_ changed by >20% from its starting value, or where a current of >100 pA was required to hold the membrane potential at −60 mV, were discarded from our analyses.

For resting membrane potential measurement, DGCs were recorded at ∼35°C in conditioned media with +15 mM NaCl or KCl, using an internal solution that contained 130 mM K-gluconate, 10 mM NaCl, 1 mM EGTA, 0.133 mM CaCl_2_, 2 mM MgCl_2_, 10 mM HEPES, 3.5 mM MgATP, and 1 mM NaGTP (pH 7.4, 290 mOsm, LJP ∼15 mV). Resting membrane potential was either estimated in the on-cell configuration, using the reversal potential of somatic voltage-gated potassium currents measured in response to a depolarizing ramp stimulus (Verheugen et al., 1999), or in whole-cell current clamp at *I* = 0 immediately after membrane rupture. These methods gave statistically indistinguishable results (two-way ANOVA; treatment, F_1,31_ = 147.3, p < 0.0001; recording mode, F_1,31_ = 0.5, p = 0.51; interaction, F_1,31_ = 0.59, p = 0.45), and were grouped.

For live AIS imaging prior to all other electrophysiological recordings, we utilized the mouse-anti-pan-neurofascin antibody (NF-ext, A12/18, Neuromab). Pre-labeling, neurons were incubated for 5 min at 37°C in 50: 50 conditioned media: fresh Neurobasal, with 50 μM APV (Sigma) to protect against cell death (Hogins et al., 2011). They were then transferred to primary labeling solution, NF-ext (1:200) and 50 μM APV in fresh Neurobasal, for 3 min at 37°C. After three washes in Neurobasal, we labeled with secondary antibody (anti-mouse Alexa 488, 1:500, Invitrogen) for 10 s at RT. After three further Neurobasal washes, neurons were transferred to HEPES-buffered saline (HBS) for recording (see below).

For AP recordings, coverslips were treated with either + 15 mM NaCl or + 15 mM KCl for 3 hr before being live-labeled for NF-ext and placed in identical HBS extracellular solution (pH 7.4, ∼290 mOsm, LJP ∼15 mV) containing 136 mM NaCl, 2.5 mM KCl, 10 mM HEPES, 10 mM d-glucose, 2 mM CaCl_2_, 1.3 mM MgCl_2_, 0.01 mM SR-95531 (gabazine, Sigma), 0.02 mM NBQX, and 0.025 mM APV. In pro-PKA recording conditions, HBS also contained 1 μM cyclosporin A (Abcam), 10 μM forskolin (Cambridge Bioscience), and 4 nM tautomycetin (R&D Systems). In current-clamp mode under full bridge balance, evoked spikes were measured with *V*_hold_ set to −60 ± 3 mV. For AP waveform measures, we injected 10-ms current steps of increasing amplitude with 2-s inter-sweep interval until reaching the current threshold at which the neuron reliably fired an AP (V_m_ > 0 mV). For multiple spiking measures, we injected 500-ms current steps of increasing amplitude with 2-s inter-sweep interval until the neuron passed its maximum spike number.

For assessment of whole-cell sodium current, live-labeled DGCs were recorded in a reversed [Na^+^] gradient (Garrido et al., 2003). Extracellular solution contained 90 mM ChCl, 30 mM HEPES, 15 mM TEA-Cl, 5 glucose, 5 mM Na-acetate, 2.5 mM MgCl2, 1 mM KCl, 0.2 mM CdCl_2_ (pH ∼7.3 with TMA-OH, 265 mOsm; pro-PKA conditions as above). Intracellular solution contained 100 mM NaF, 30 mM NaCl, 20 mM CsF, and 5 mM HEPES (pH ∼7.3 with CsOH, 285 mOsm, LJP ∼7 mV). *R*_*s*_ was compensated ∼60%. For activation, cells were held at −80 mV before applying 20-ms depolarizing steps that increased by 4 mV. For steady-state inactivation, cells were held for 500 ms at a series of potentials from −80 mV in 4-mV increments before being stepped to the 0-mV test potential.

In on-cell recordings, voltages are displayed relative to resting potential and traces are inverted as per convention. For somatic sodium current, live-labeled DGCs were recorded in HBS with synaptic receptor antagonists. Pipettes did not differ between treatment groups (+15 mM NaCl 4.27 ± 0.15 MΩ; +15 mM KCl 4.43 ± 0.21 MΩ; t test, t_49_ = 0.63, p = 0.53) and contained HBS with 10 mM TEA, 5 μM 4-AP, and 200 μM CdCl_2_ (pH 7.3, 290 mOsm, LJP ∼0 mV). Once a supra-gigaseal had been obtained (seal resistance; +15 mM NaCl 8.02 ± 0.36 GΩ; +15 mM KCl 8.08 ± 0.58 GΩ; t test, t_49_ = 0.10, p = 0.92), DGCs were held at −80 mV in on-cell mode before applying 20-ms steps over a range of −80 mV to +120 mV.

For somatic potassium current, pipettes did not differ between treatment groups (+15 mM NaCl 5.54 ± 0.32 MΩ; +15 mM KCl 5.62 ± 0.27 MΩ; Mann-Whitney test, p = 0.62, n = 57) and contained HBS plus 1 μM TTX (pH 7.3, 290 mOsm, LJP ∼0 mV). After formation of a supra-gigaseal (seal resistance, +15 mM NaCl 12.2 ± 0.5 GΩ; +15 mM KCl 11.5 ± 0.7 GΩ; t test, t_55_ = 0.90, p = 0.37), cells were held at −40 mV in on-cell mode before applying 200-ms depolarizing steps that increased incrementally by 16 mV. Inter-sweep interval was 10 s to allow for current de-inactivation. To reduce the contribution from fast-inactivating *I*_A_ currents, we added a 30-ms depolarization to +60 mV prior to each depolarizing step (Bekkers, 2000, Misonou et al., 2004).

Exported traces were analyzed using custom-written MATLAB routines. To determine voltage threshold, 5-μs sample interval recordings of spikes fired at threshold 10-ms current injection were smoothed using a 20-point (100-μs) sliding filter, before differentiation for dVdt. Voltage threshold was taken as the unsmoothed potential at which dVdt first passed 10 V/s. Spike width was measured at the midpoint between voltage threshold and maximum voltage. Rheobase and AHP were both measured from responses to 500-ms current injection, the latter from the local voltage minimum following the first spike fired at rheobase. For comparison of spike number and tonic depolarization across different injected current densities, measures were averaged for each cell in bins of 0.5 pA/pF width, and subjected to mixed model analysis for repeated-measures data with current density bin as the within-subject variable. Current density versus tonic depolarization curves for each neuron were fitted with single exponential functions for determination of plateau and rate constant (k) parameters. All on-cell and whole-cell voltage-clamp recordings were subject to P4 subtraction, with response amplitudes determined from the peak leak-subtracted current at each test voltage. Voltage versus current density activation curves for each cell were fitted with a straight line from −32 to +40 mV (r^2^ ≥ 0.94) to determine the slope of the relationship. Normalized chord conductance (*g*/*g*_max_) values for activation curves were calculated based on an *E*_Na_ of −76 mV; these, along with normalized current density (*I*/*I*_max_) values for inactivation curves, were fitted with standard Boltzmann equations (r^2^ ≥ 0.95; Prism, GraphPad) to obtain V_50_ and slope factors.

### Statistics

Sample distributions were assessed for normality using the D’Agostino and Pearson omnibus test and compared using parametric or non-parametric tests accordingly using Prism or SPSS (IBM). All tests were two tailed with the level of significance at 0.05.

## Author Contributions

M.D.E., A.S.D., D.L.H.K., and M.S.G. designed experiments. M.D.E., A.S.D., D.L.H.K., and M.S.G. performed activity manipulations and immunohistochemical labeling. M.D.E., A.S.D., S.E.T., and M.S.G. obtained electrophysiological recordings. All authors analyzed data and discussed the results, and M.D.E., A.S.D., and M.S.G. wrote the paper.

## Supplemental Information

Document S1. Figures S1–S7 and Tables S1–S3

Document S2. Article plus Supplemental Information
