# Supplementary material for: Rapid Modulation of Axon Initial Segment Length Influences Repetitive Spike Firing
Source: Cell Rep. 2015 Oct 29;13(6):1233–45. doi: 10.1016/j.celrep.2015.09.066 (PMC4646840; doi:10.1016/j.celrep.2015.09.066)
Supplement: Document S1. Figures S1–S7 and Tables S1–S3 [file mmc1.pdf]

Cell Reports

Supplemental Information

Rapid Modulation of Axon Initial Segment Length Influences Repetitive Spike Firing

Mark D. Evans, Adna S. Dumitrescu, Dennis L.H. Kruijssen, Samuel E. Taylor, and Matthew S. Grubb

Supplemental Data

Figure S1

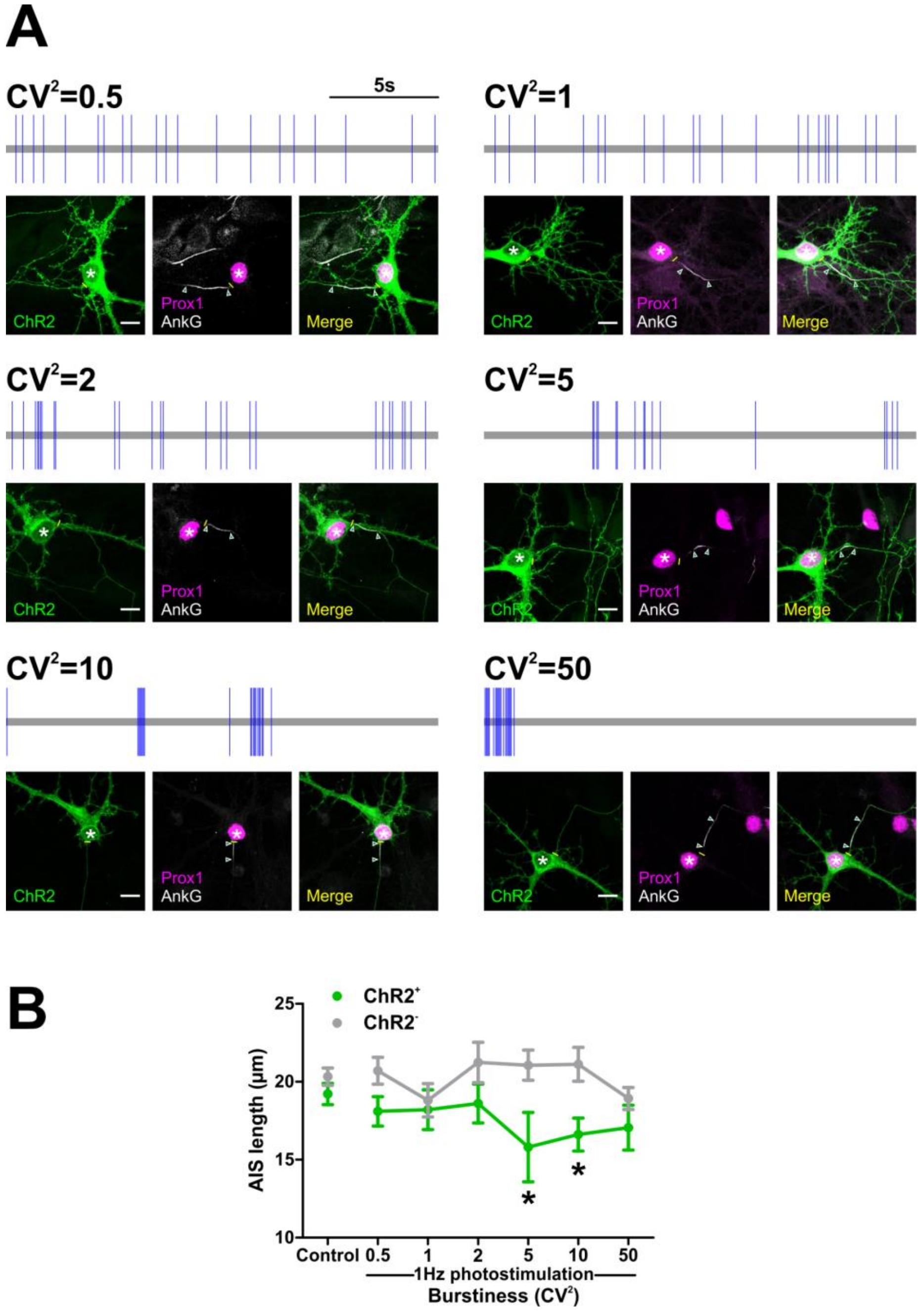

Figure S1. Rapid AIS plasticity is dependent on burstiness in naturalistic optogenetic stimulation patterns, related to Figure 1.

(A) Schematic of photostimulus (top). Each blue bar represents a single 5 ms flash. Inter-flash intervals were sampled randomly from a negative binomial distribution with mean frequency 1 Hz and co-efficient of variation (CV^2) as depicted for each group. Maximum intensity projections (bottom) show sparsely channelrhodopsin-2 (ChR2)-expressing cultures co-stained for Prox1 and AnkG after 3 h of each patterned photostimulus. Asterisks mark soma of $ChR2^+$ DGCs; lines show axon start, arrowheads show DGC AIS start and end positions, scalebar shows 10 µm.

(B) Mean ± SEM of AIS length in $ChR2^+$ and $ChR2^-$ DGCs in all stimulus groups. Control cells were not photostimulated. Bonferroni post-test after 2-way ANOVA; *, $p < 0.05$.

Figure S2

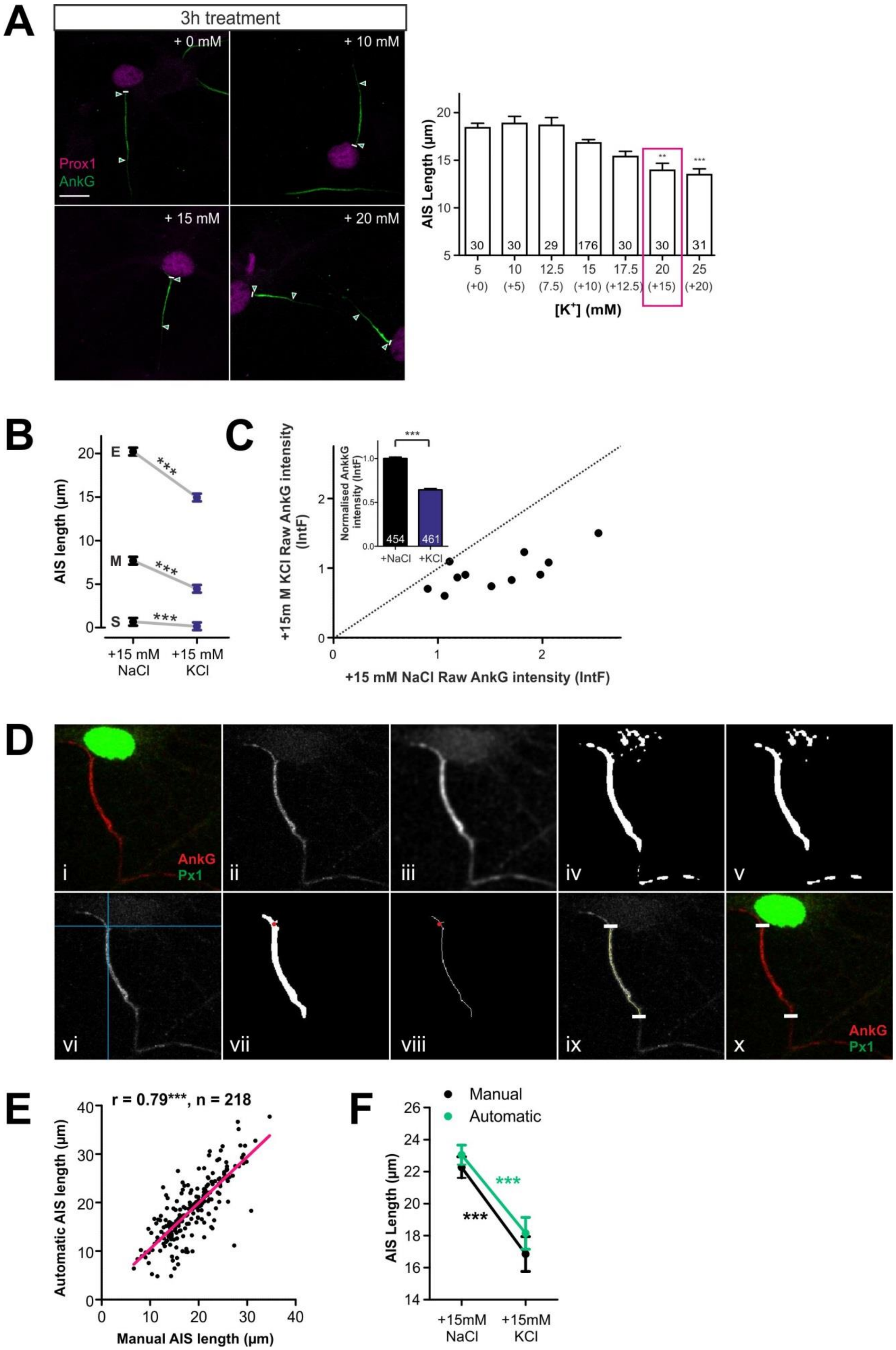
A
3h treatment
+ 0 mM
+ 10 mM
+ 15 mM
+ 20 mM
Prox1
AnkG
AIS Length (µm)
[K+] (mM)
5 (+0)
10 (+5)
12.5 (7.5)
15 (+10)
17.5 (+12.5)
20 (+15)
25 (+20)
30
30
29
176
30
30
31
B
AIS length (µm)
E
M
S
+15 mM NaCl
+15 mM KCl
C
+15m M KCl Raw AnkG intensity (IntF)
+15 mM NaCl Raw AnkG intensity (IntF)
Normalised AnkG intensity (IntF)
454
461
+NaCl
+KCl
D
AnkG
Px1
i
ii
iii
iv
v
vi
vii
viii
ix
x
E
r = 0.79***, n = 218
Automatic AIS length (µm)
Manual AIS length (µm)
F
Manual
Automatic
AIS Length (µm)
+15mM NaCl
+15mM KCl

Figure S2. Characterising rapid shortening of axonal AnkG distributions, related to Figure 2.

(A) Maximum intensity projections (left) of DGCs co-labelled for Prox1 and AnkG after 3 h treatment with different $[K^+]_o$. Plot (right) shows mean + SEM for AIS length. Magenta rectangle shows KCl concentration chosen for future experiments. Dunn's post-test versus +0 mM after Kruskal-Wallis 1-way ANOVA; **, $p < 0.01$; ***, $p < 0.001$.

(B) Mean ± SEM of AnkG-defined AIS start (S), maximum fluorescence (M) and end (E) positions after 3 h +15 mM NaCl or KCl treatment. Mann-Whitney test; ***, $p < 0.001$.

(C) Scatter plot of raw integrated AIS AnkG immunofluorescence intensity (IntF) after 3 h +15 mM NaCl versus KCl treatments. Each dot shows the mean for a single experiment; dotted line shows unity. Inset shows mean + SEM IntF across all experiments in both groups, normalised to the mean NaCl value in each experiment. Numbers in bars show cell numbers in each group. Mann-Whitney test; ***, $p < 0.001$.

(D) Independent quantification of AIS length. A maximum intensity projection of DGC AnkG label (i,ii) is smoothed using a 2D Gaussian (iii). The image is thresholded (iv) and morphologically opened and closed (v) to remove discontinuities before the user indicates the AIS start position (vi, crosshairs; vii, red dot). The single connected element closest to this point is thinned to produce an 'AIS skeleton' one pixel wide (viii) which is fitted with a 2D cubic smoothing spline (ix, yellow line) whose start and end positions define AIS length (ix,x, white lines).

(E) Scatter plot of DGC AIS lengths measured with manual and automated methods. Each dot shows one cell. Pearson correlation; ***, $p < 0.001$.

(F) Mean ± SEM of AIS length measured using both methods in control and depolarised DGCs. Bonferroni post-test after repeated-measures 2-way ANOVA; ***, $p < 0.001$.

Figure S3

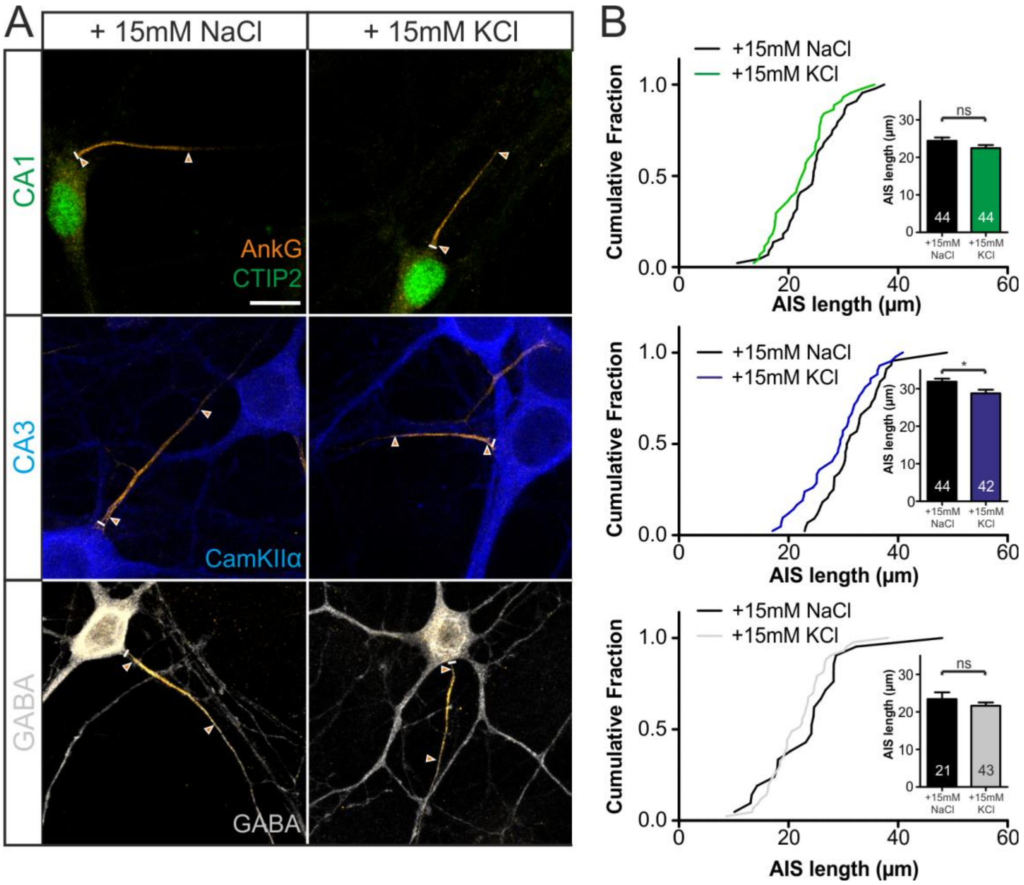

Figure S3. Cell-type specificity of rapid AIS plasticity, related to Figure 2.

(A) Maximum intensity projections of neurons treated for 3 h with +15 mM NaCl or KCl, then stained for AnkG plus hippocampal cell-type markers to identify CA1, CA3, and GABAergic neurons (see Experimental Procedures). Lines show axon start, arrowheads show AIS start and end positions, scalebar shows 10 µm.

(B) Cumulative fraction and (inset) mean + SEM of AIS length in both groups for each cell-type. Numbers in bars show cell number in each group. Unpaired t-test (CA1, CA3) or Mann-Whitney test (GABA); *, $p < 0.05$; ns, non-significant.

Figure S4

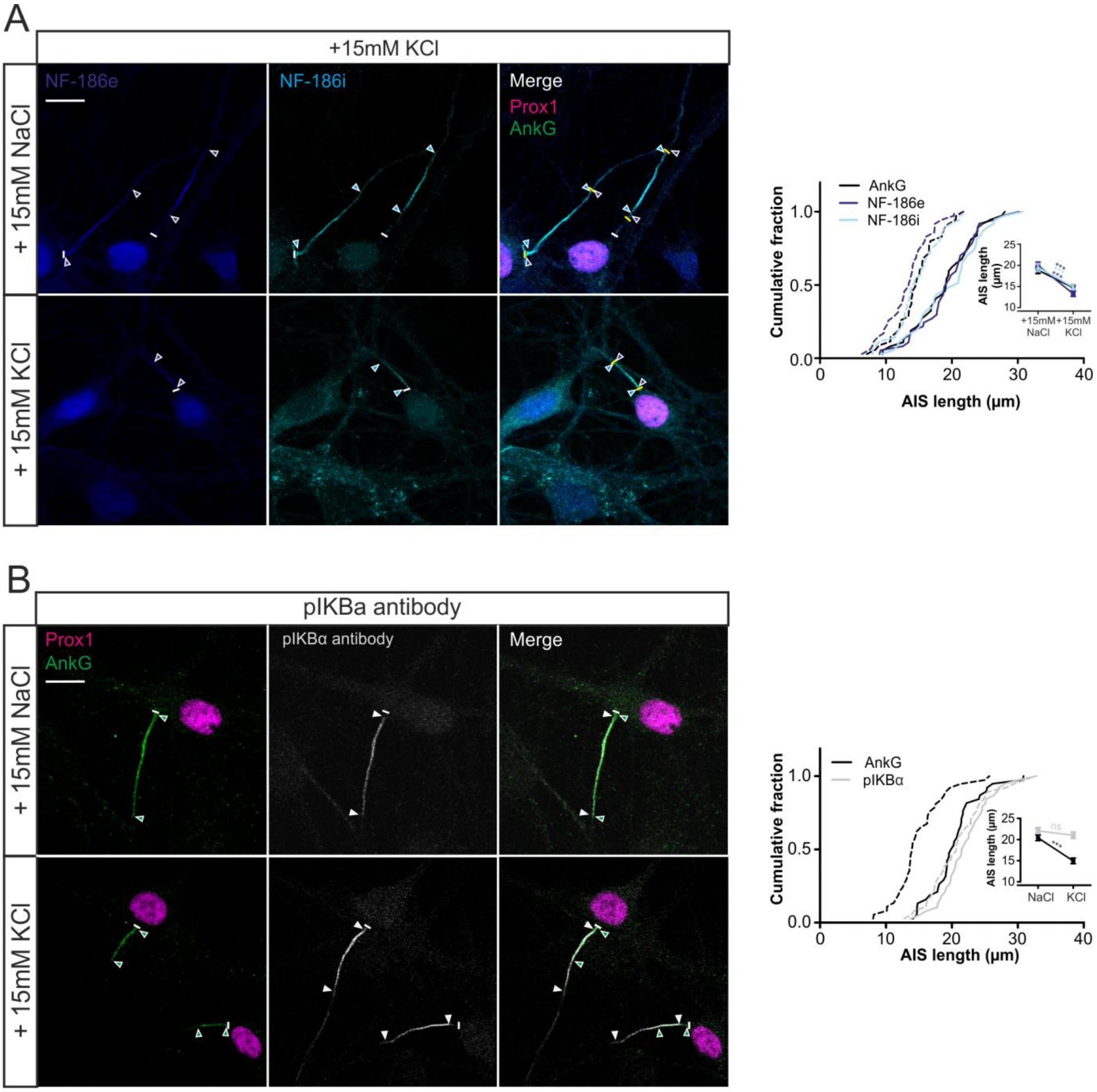

Figure S4. Differential effects of rapid plasticity on AIS components, related to Figure 3.

(A) Maximum intensity projections (left) of neurons treated for 3 h with +15 mM NaCl or KCl, then stained for AnkG and prox1, plus extracellular and intracellular pan-neurofascin (NF-ext and NF-int, respectively). White lines show axon start, arrowheads show AIS start and end positions for NF-ext (blue) and NF-int (cyan), yellow lines show AIS start and end positions for AnkG, scalebar shows 10 µm. Plots (right) show cumulative fraction and (inset)

mean ± SEM of AIS lengths in each group. Bonferroni post-test after repeated-measures 2-way ANOVA; ***, $p < 0.001$.

(B) Maximum intensity projections (left) of neurons treated for 3 h with +15 mM NaCl or KCl, then stained for AnkG and prox1, plus the 'pIκBα' antibody. White lines show axon start, arrowheads show AIS start and end positions for AnkG (green) and pIκBα (white), scalebar shows 10 µm. Plots (right) show cumulative fraction and (inset) mean ± SEM of AIS lengths in each group. Bonferroni post-test after repeated-measures 2-way ANOVA; ***, $p < 0.001$; ns, non-significant.

Figure S5

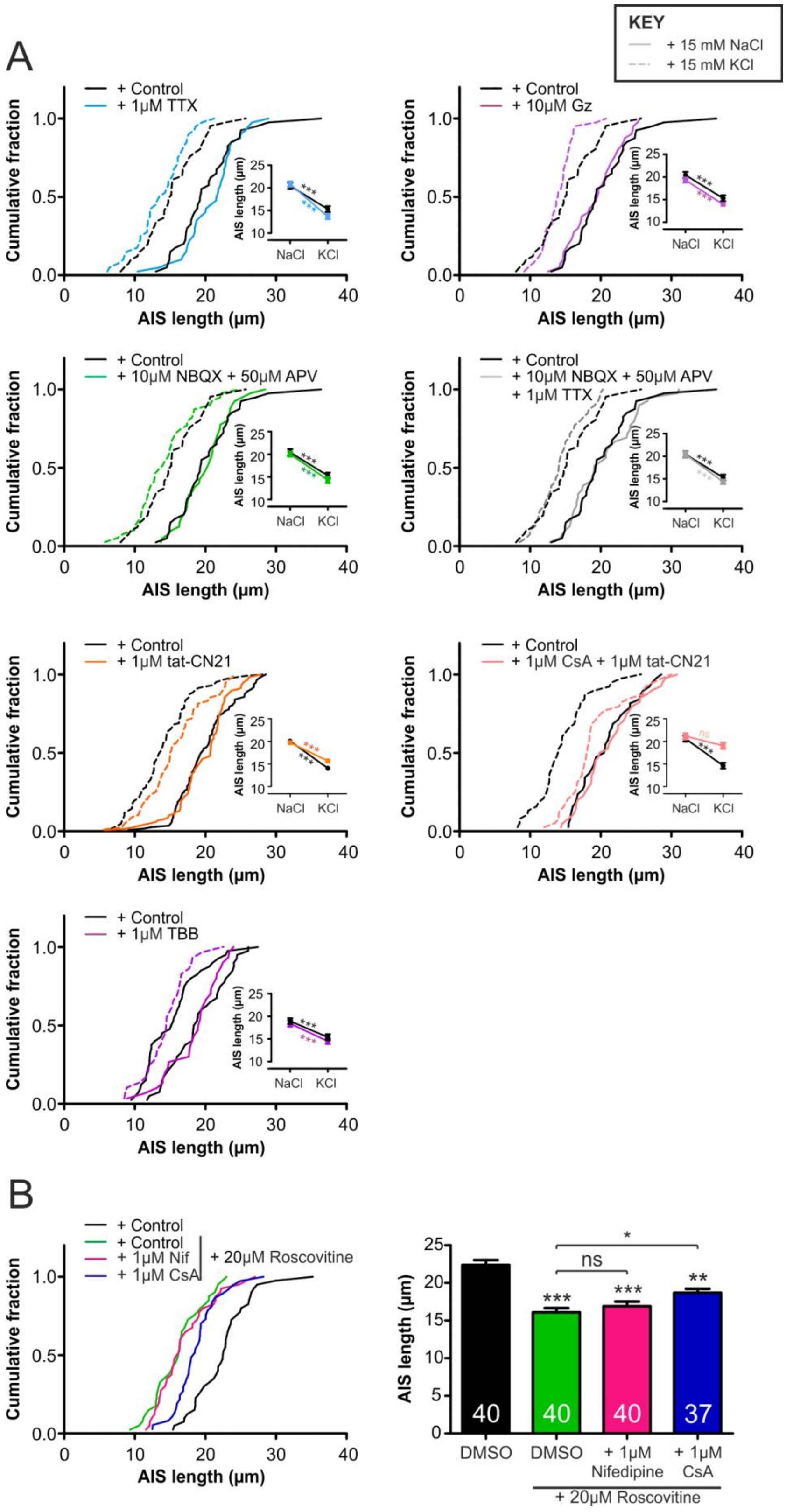
KEY
+ 15 mM NaCl
+ 15 mM KCl
A
+ Control
+ 1μM TTX
+ 10μM Gz
+ 10μM NBQX + 50μM APV
+ 10μM NBQX + 50μM APV + 1μM TTX
+ 1μM tat-CN21
+ 1μM CsA + 1μM tat-CN21
+ 1μM TBB
Cumulative fraction
AIS length (μm)
NaCl
KCl
ns
B
+ Control
+ 1μM Nif
+ 1μM CsA
+ 20μM Roscovitine
DMSO
+ 1μM Nifedipine
+ 1μM CsA
40
40
40
37

Figure S5. Signalling pathways in rapid AIS shortening, related to Figure 5.

(A) Plots show cumulative fraction and (inset) mean ± SEM of AIS lengths following 3 h +15 mM NaCl or KCl treatment in the presence of various pharmacological agents. Gz, gabazine; Bonferroni post-test after 2-way ANOVA; **, $p < 0.01$; ***, $p < 0.001$; ns, non-significant.

(B) Cumulative fraction plot (left) of AIS length after 24 h CDK5 inhibition in the presence of L-type calcium channel or calcineurin block. Nif, nifedipine; CsA, cyclosporin A. Mean + SEM plot (right) of AIS length in each group. Numbers in bars show cell numbers in each group. Tukey post-hoc test following one-way ANOVA; *, $p < 0.05$; **, $p < 0.01$; ***, $p < 0.001$.

Figure S6

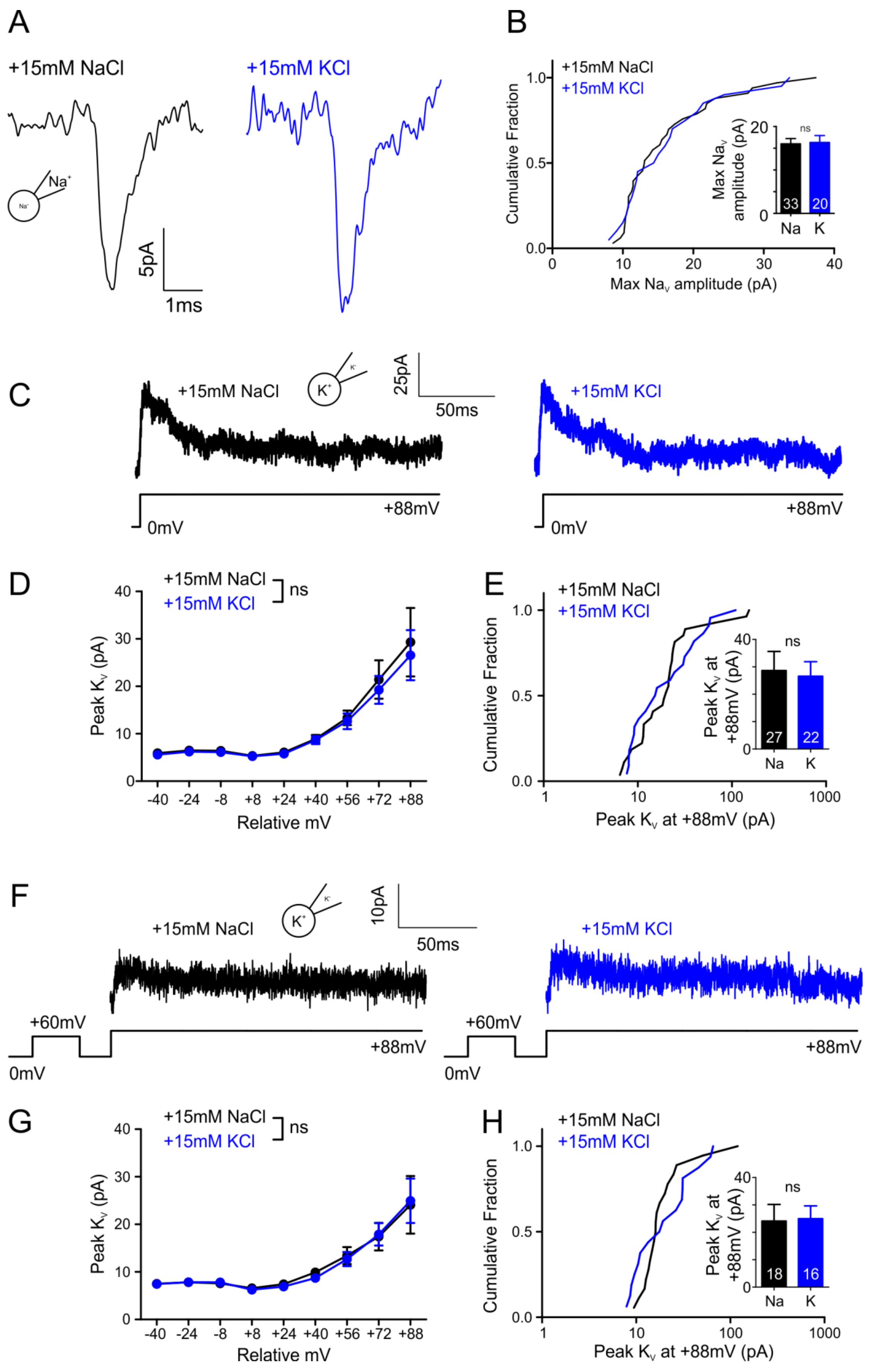
A
+15mM NaCl
+15mM KCl
Na+
5pA
1ms
B
+15mM NaCl
+15mM KCl
Cumulative Fraction
Max Nav amplitude (pA)
ns
33
20
Na
K
C
+15mM NaCl
+15mM KCl
K+
25pA
50ms
0mV
+88mV
D
+15mM NaCl
+15mM KCl
ns
Peak Kv (pA)
Relative mV
E
Cumulative Fraction
Peak Kv at +88mV (pA)
27
22
F
+15mM NaCl
+15mM KCl
10pA
50ms
+60mV
0mV
+88mV
G
+15mM NaCl
+15mM KCl
ns
Peak Kv (pA)
Relative mV
H
Cumulative Fraction
Peak Kv at +88mV (pA)
18
16

Figure S6. Somatic Na_V and K_V current amplitude is unchanged following 3 h depolarisation, related to Figure 6.

(A) Example maximum amplitude somatic Na_V currents, recorded in cell-attached mode after increasingly depolarised voltage steps from rest in 3 h control and depolarised neurons.

(B) Cumulative fraction and (inset) mean + SEM of maximum somatic Na_V amplitude in both groups. Numbers in bars show cell numbers in each group. Mann-Whitney test; ns, non-significant.

(C) Example ensemble somatic K_V currents, recorded in cell-attached mode after depolarisation to +88 mV above rest after 3 h +15 mM NaCl or KCl treatment.

(D) Mean ± SEM peak K_V amplitude at different relative voltages for both treatment groups. Effect of treatment group in repeated-measures 2-way ANOVA; ns, non-significant.

(E) Cumulative fraction and (inset) mean + SEM plots for peak somatic K_V amplitude at +88 mV. Numbers show cell numbers in each group. Mann-Whitney test; ns, non-significant.

(F) Example somatic cell-attached K_V currents biased towards I_K, after inactivation of I_A components with a brief depolarisation pre-pulse.

(G) Mean ± SEM peak I_K-biased K_V amplitude at different relative voltages for both treatment groups. Effect of treatment group in repeated-measures 2-way ANOVA; ns, non-significant.

(H) Cumulative fraction and (inset) mean + SEM plots for peak I_K-biased somatic K_V amplitude at +88 mV. Numbers show cell numbers in each group. Mann-Whitney test; ns, non-significant.

Figure S7

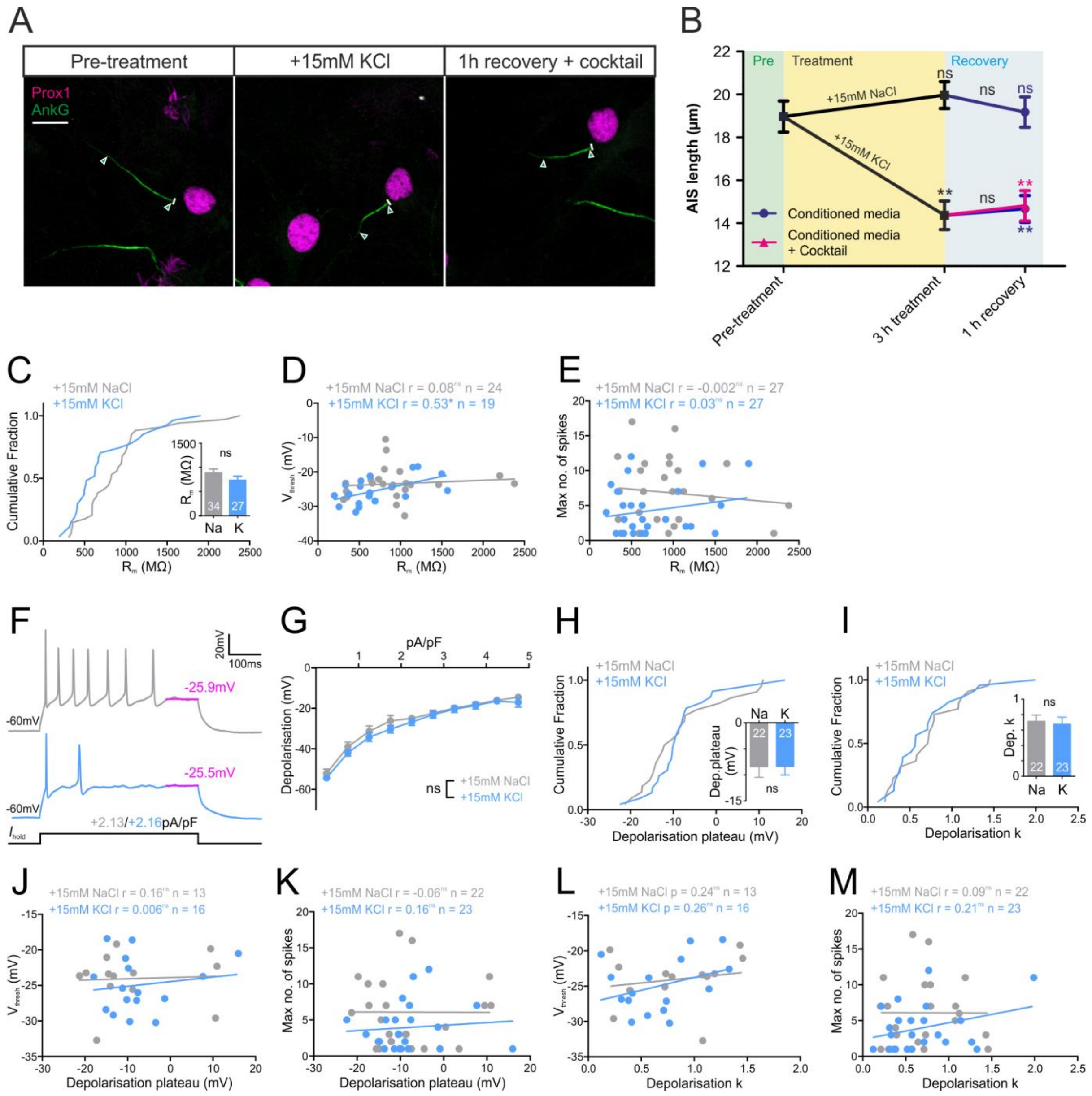

Figure S7. AIS shortening, membrane resistance, and tonic responses to depolarising current in pro-PKA conditions, related to Figure 7.

(A) Pro-PKA conditions do not reverse rapid AIS shortening. Maximum intensity projections of neurons pre-depolarisation, after 3 h +15 mM KCl treatment, and after an additional 1 h recovery in a pro-PKA cocktail, co-labelled for AnkG and prox1. Lines show axon start, arrowheads show AIS start and end positions, scalebar shows 10 µm.

(B) Mean ± SEM AIS length in all groups. Dunn's post-test following Kruskal-Wallis one-way ANOVA; symbols above data points show tests versus pre-treatment group; symbols above connecting lines show tests between connected groups; **, $p < 0.01$; ns, non-significant.

(C) Cumulative fraction plot and (inset) mean + SEM of membrane resistance (R_m) in pro-PKA conditions after 3 h +15 mM NaCl or KCl treatment. Numbers in bars show cell numbers in each group. Mann-Whitney test; ns, non-significant.

(D) & (E) Scatter plots of R_m versus voltage threshold (D) and maximum spike number (E) in both treatment groups. Each dot shows one cell; lines show best fit linear regression. Spearman correlation; *, $p < 0.05$; ns, non-significant.

(F) Example current-clamp traces showing tonic depolarisation (magenta) in control and 3 h depolarised neurons at ~2.15 pA/pF 500 ms current injection.

(G) Mean ± SEM tonic depolarisation at different amplitudes of current injection for both groups. Mixed model effect of treatment group; ns, non-significant.

(H) & (I) Cumulative fraction and (inset) mean & SEM plots for parameters of exponential fits to current versus depolarisation response curves in individual neurons: plateau depolarisation (dep.; H) and rate constant k (I). Numbers in bars show cell numbers in each group. Mann-Whitney test; ns, non-significant.

(J) to (M) Scatter plots of fitted tonic depolarisation parameters versus voltage threshold (J, L) and maximum spike number (K, M) in both treatment groups. Each dot shows one cell; lines show best fit linear regression. Spearman correlation; *, $p < 0.05$; ns, non-significant.

Table S1.

Parameter	Mean ± SEM (n)		Group comparison	Correlation with AIS Length	
	+15 mM NaCl	+15 mM KCl		+15 mM NaCl	+15 mM KCl
R_m (MΩ)	911 ± 92 (35)	679 ± 50 (33)	***U = 413 p = 0.044***	Sr = -0.24 p = 0.17	Sr = -0.014 p = 0.94
C_m (pF)	34.9 ± 1.4 (35)	33.5 ± 1.2 (33)	t = 0.75 p = 0.46	***Pr = 0.36 p = 0.033***	Pr = 0.18 p = 0.31
V_{thresh} (mV)	-24.3 ± 0.7 (34)	-25.4 ± 0.7 (32)	t = 1.07 p = 0.29	***Pr = -0.39 p = 0.022***	Pr = -0.15 p = 0.41
V_{max} (mV)	32.8 ± 1.9 (34)	30.8 ± 2.1 (32)	t = 0.72 p = 0.47	Pr = 0.15 p = 0.40	Pr = -0.039 p = 0.83
Max dVdt (V/s)	99.8 ± 8.1 (34)	89.0 ± 9.5 (32)	t = 0.87 p = 0.39	Pr = 0.17 p = 0.34	Pr = 0.022 p = 0.91
AP width (ms)	1.68 ± 0.10 (34)	1.85 ± 0.11 (32)	U = 435 p = 0.16	Sr = -0.11 p = 0.52	Sr = 0.11 p = 0.55
Rheobase (pA/pF)	0.75 ± 0.12 (16)	1.10 ± 0.18 (16)	*U = 80 p = 0.073*	Sr = -0.33 p = 0.22	Sr = 0.27 p = 0.32
AHP (mV)	-40.6 ± 1.6 (16)	-41.2 ± 1.7 (16)	U = 106 p = 0.42	*Sr = 0.46 p = 0.072*	Sr = 0.26 p = 0.34
Max no. of spikes	5.29 ± 0.99 (24)	4.15 ± 0.63 (27)	U = 297 p = 0.61	Sr = 0.003 p = 0.99	Sr = -0.27 p = 0.18
Dep. plateau (mV)	-9.84 ± 1.54 (15)	-9.96 ± 1.80 (23)	t = 0.044 p = 0.97	Pr = 0.21 p = 0.46	Pr = 0.004 p = 0.99
Dep. K	0.73 ± 0.09 (15)	0.70 ± 0.07 (23)	t = 0.26 p = 0.80	Pr = -0.021 p = 0.94	Pr = 0.11 p = 0.63

Supplemental Table 1. Physiological parameters of DGCs recorded under baseline conditions, related to Figure 6. Group comparisons show results of Mann-Whitney U test or unpaired t-test, and correlations report Spearman's r (Sr) or Pearson's r (Pr), for non-parametric and parametric datasets, respectively. Italics highlight tests where $p < 0.1$; bold highlights tests where $p < 0.05$; Dep., depolarisation.

Table S2.

Parameter	Mean ± SEM (n)		Group comparison	Correlation with AIS Length	
	**+15 mM NaCl**	**+15 mM KCl**		**+15 mM NaCl**	**+15 mM KCl**
R_m (MΩ)	886 ± 80 (34)	732 ± 86 (27)	U = 352 p = 0.12	*Sr = -0.33 p = 0.053*	Sr = -0.02 p = 0.92
C_m (pF)	31.3 ± 1.2 (35)	33.8 ± 1.3 (27)	U = 359 p = 0.11	*Sr = 0.30 p = 0.078*	Sr = -0.03 p = 0.89
V_{thresh} (mV)	-23.4 ± 0.9 (24)	-25.3 ± 0.9 (19)	U = 167 p = 0.14	***Sr = -0.44 p = 0.03***	***Sr = -0.64 p = 0.003***
V_{max} (mV)	30.4 ± 2.5 (24)	30.3 ± 3.3 (19)	t = 0.04 p = 0.97	Pr = 0.31 p = 0.14	Pr = 0.38 p = 0.11
Max dVdt (V/s)	112 ± 12 (24)	120 ± 17 (19)	t = 0.39 p = 0.70	Pr = 0.29 p = 0.17	*Pr = 0.40 p = 0.09*
AP width (ms)	1.59 ± 0.14 (24)	1.46 ± 0.14 (19)	U = 205 p = 0.58	Sr = -0.28 p = 0.18	Sr = -0.31 p = 0.20
Rheobase (pA/pF)	1.29 ± 0.14 (26)	1.70 ± 0.25 (23)	U = 248 p = 0.31	Sr = -0.06 p = 0.76	Sr = 0.07 p = 0.75
AHP (mV)	-40.0 ± 1.1 (26)	-39.0 ± 1.2 (23)	U = 290 p = 0.86	Sr = 0.15 p = 0.46	Sr = 0.03 p = 0.89
Max no. of spikes	6.96 ± 0.87 (28)	4.26 ± 0.68 (27)	***t = 2.43 p = 0.019***	***Pr = 0.52 p = 0.005***	***Pr = 0.58 p = 0.001***
Dep.plateau (mV)	-8.38 ± 2.06 (22)	-8.33 ± 1.69 (23)	U = 235 p = 0.69	Sr = 0.008 p = 0.97	Sr = 0.31 p = 0.15
Dep. K	0.71 ± 0.08 (22)	0.68 ± 0.09 (23)	U = 231 p = 0.62	Sr = -0.22 p = 0.33	Sr = -0.07 p = 0.74

Supplemental Table 2. Physiological parameters of DGCs recorded under pro-PKA conditions, related to Figure 7. Group comparisons show results of Mann-Whitney U test or unpaired t-test, and correlations report Spearman's r (Sr) or Pearson's r (Pr), for non-parametric and parametric datasets, respectively. Italics highlight tests where $p < 0.1$; bold highlights tests where $p < 0.05$; Dep., depolarisation.

Table S3

Antigen	Species	Isotype	Supplier	Working dilution
Ankyrin-G	Mouse (monoclonal)	IgG2a	NeuroMab (N106/36)	1:500
αCaMKII	Mouse (monoclonal)	IgG1	Millipore	1:1000
CTIP2	Rat (monoclonal)		Abcam	1:1000
GABA	Rabbit (polyclonal)		Sigma	1:1000
$Na_V1.2$	Mouse (monoclonal)	IgG2a	NeuroMab (K69/3)	1:1000
Pan-Na_V	Mouse (monoclonal)	IgG1	Sigma	1:100
Pan-Neurofascin (external)	Mouse (monoclonal)	IgG2a	NeuroMab (A12/18)	1:500
Pan-Neurofascin (internal)	Mouse (monoclonal)	IgG1	NeuroMab (L11A/41)	1:500
'pIκBα'	Rabbit (monoclonal)		Cell Signaling (14D4)	1:500
Prox1	Rabbit (polyclonal)		Sigma	1:1000

Supplemental Table 3. Primary antibodies, related to Experimental Procedures.
